# Changes of structural, magnetic and spectroscopic properties of microencapsulated iron sucrose nanoparticles in saline

**DOI:** 10.3762/bjnano.16.59

**Published:** 2025-06-02

**Authors:** Sabina Lewińska, Pavlo Aleshkevych, Roman Minikayev, Anna Bajorek, Mateusz Dulski, Krystian Prusik, Tomasz Wojciechowski, Anna Ślawska-Waniewska

**Affiliations:** 1 Institute of Physics, Polish Academy of Sciences, Al. Lotników 32/46, 02-668 Warsaw, Poland; 2 Silesian Center for Education and Interdisciplinary Research, University of Silesia in Katowice, 75 Pułku Piechoty 1A, 41-500 Chorzów, Poland; 3 A. Chełkowski Institute of Physics, University of Silesia in Katowice, 75 Pułku Piechoty 1, 41-500 Chorzów, Poland; 4 Institute of Materials Engineering, University of Silesia, and Silesian Center for Education and Interdisciplinary Research, 75 Pułku Piechoty 1A, 41-500 Chorzów, Poland; 5 International Research Centre MagTop, Institute of Physics, Polish Academy of Sciences, Aleja Lotników 32/46, 02-668 Warsaw, Poland

**Keywords:** calcium alginate, dissolution, iron oxyhydroxide nanoparticles, magnetic properties, saline, spectroscopy

## Abstract

The structural and physical properties of microencapsulated iron sucrose and their changes upon dissolution in saline were tested. For the undissolved sample, calcium alginate microcapsules with irregular shapes were registered via scanning electron microscopy, inside which core–shell nanoparticles were identified by transmission electron microscopy micrographs. Magnetic studies (DC and AC) performed on the undissolved sample revealed the presence of a low temperature blocking process (<*T*_B_> ≈ 10 K), and confirmed its superparamagnetic state between 70– 250 K. X-ray photoelectron spectroscopy and Raman studies showed a varied composition of the undissolved sample in which organic compounds and SiO_2_ are the major phases, while the iron phase was recognized as iron oxyhydroxide (FeOOH) (most probably the α polymorph). The dissolution procedure had significant influence on structural and physical properties of the investigated compound, such as lowering of the blocking temperature with the dissolution time. Electron paramagnetic resonance (EPR) studies performed on the completely dissolved sample revealed that some of the Fe^3+^ ions became paramagnetic, while the rest remained exchange coupled into clusters. The nonintentional manganese contamination was determined using EPR in the completely dissolved sample.

## Introduction

Hematinic are substances such as iron, vitamin B12, and folic acid that are essential in the production of blood [1]. Iron deficiency drugs, which belong to the hematinic category, are a broad class of medical products in which varied iron agents are used, very often in the form of core–shell nanoparticles, where iron ions (in the structure of iron oxide mineral) are concentrated in the core, and the shell is generally composed of carbohydrates that stabilized the entire nanoparticle [2–4]. Such a variety of the iron delivery drugs in the pharmaceutical market results from the continuous improvement of their properties, such as absorption of iron in the human body, time of iron release after administration to the patient, and shape and size of the drug for easier swallowing. Simultaneously, all these compounds due to the iron content should exhibit magnetic properties that may potentially turn out to be interesting. Moreover, since all iron deficiency agents are commercial products and are mass produced, their properties can be expected to be stable and reproducible. From this perspective, iron deficiency drugs represent an infinite source of samples to study the behavior of iron compounds in a wide variety of carbohydrate and polymeric surroundings.

A typical superparamagnetic behavior was recognized for several iron deficiency drugs with different iron agents, such as ferumoxytol [5], iron isomaltoside [5], and iron sucrose [6–8]. Such behavior is expected, and not surprising, as in most cases iron agents are systems of noninteracting magnetic nanoparticles. However, more complex process such as two superimposed relaxation processes observed for ferric carboxymaltose are also reported [9]. Conversely, ferrous gluconate, according to [10], exhibit paramagnetic behavior, and the possibility of a short-range magnetic order at low temperatures is only suggested, which means that the iron ions are sufficiently well separated from each other and not magnetically interacting.

There are many others literature examples of studies of chemical and physical properties on iron deficiency drugs and their usage in agents [2,7–8,11–15]. However, in this work the whole attention is focused on the iron deficiency agent called iron sucrose. The original iron sucrose available on the market is the intravenous Venofer^®^ (Vifor, Switzerland), whose physical and chemical properties are thoroughly tested and described in the literature [5–9,13–14].

Generally, iron sucrose originating from Venofer^®^ has a core–shell nanoparticle form, where the shell consists of sucrose, while the core is iron(III) oxide mineral. The average diameter of iron sucrose nanoparticles included in Venofer^®^ was estimated as ≈7 nm, whose core diameter is 3 nm [2,7–8,13]. The elemental analysis for a single iron sucrose nanoparticle with a 3 nm core [7], showed that the core consists of 416 akaganeite (β-FeOOH) and is surrounded by 24 sucrose molecules. However, some studies have shown that iron sucrose contains ferrihydrite [4,6], 2-line ferrihydrite and akaganeite together [13], or even lepidocrocite [15]. Such discrepancies are mainly a consequence of difficulties with analysis of structural and morphological measurement results (e.g., signal obtained in X-ray diffraction (XRD) experiments is dominated by the carbohydrate). This results in very broad, hard to interpret, or even uninterpretable lines (see XRD patterns in [6–8,13,15]). Generally, it can be assumed that the iron sucrose core is composed of iron oxyhydroxide (FeO(OH)), and it is very likely that the structure is amorphous rather than crystalline [15]. Regarding the magnetic properties of FeO(OH), it exhibits an antiferromagnetic arrangement with different Néel temperatures (400 K, 250–300 Κ, and 77 K for α, β, and γ polymorphs, respectively), while in the case of ferrihydrite, which is in fact an unstable form of FeO(OH) and exists only as a nanomaterial, a canted antiferromagnetic structure is expected [16].

There are many other iron deficiency drugs based on iron sucrose that differ from Venofer^®^ in their manufacturing process, which translates into different final forms of the drug [17–18]. One of them is the diet supplement called Ferisan (Solinea Company, Poland), which is distinguished from Venofer^®^, in the pill form. Ferisan is a rather new product on the market, whose iron sucrose content has a curious form (i.e., the so-called AB-Fortis iron (Biotics)), and to our knowledge, its magnetic properties are still unexplored. Given that there is a dependence between the manufacturing process and the structure of the iron sucrose obtained [17–18], the investigation of AB-Fortis iron seems to be relevant. In this context, the AB-Fortis iron was selected as the main object of research (as the experimental sample) and an in-depth analysis of its physical properties, which in the current state of knowledge still remain undefined, is the main subject of this work. It should be stressed that the studies performed here did not have any commercial funding. An additional purpose was to analyze the morphological and structural changes of AB-Fortis iron during the dissolution process in saline. In this case, the goal was to provide information about the iron release process from such microcapsules based on changes in physical properties.

In order to carry out this study, a multi-technique analysis of undissolved and dissolved compounds was performed, including microstructure studies using scanning electron microscopy (SEM) and transmission electron microscopy (TEM). Composition studies using XRD, magnetic properties using dc and ac magnetometry, and extensive spectral analysis using Fourier-transform infrared spectroscopy (FTIR), Raman, and electron paramagnetic resonance (EPR) were also performed.

Considering that the AB-Fortis iron contains nonmagnetic pharmaceutical additives, it can be expected that the contribution from these additional substances will be significant in all obtained results. As discussed later in the text, this issue complicates the analysis of the obtained results; however, it does not constitute an obstacle to the analysis of the physical properties of iron sucrose.

The results from such in-depth analysis of the physical properties are important to elucidate the following issues: (i) the precise description of the interior structure of AB-Fortis iron, (ii) to gain knowledge about the magnetic coupling between Fe ions, (iii) the analysis of interactions between the magnetic core and the sucrose shell and their impact on core surface magnetic moments, (iv) the impact of the core surface and nonmagnetic additives on the saturation magnetization of the investigated compound [19], and (v) the consequences of the dissolution process in terms of microstructural changes and interactions between iron ions.

The paper is organized as follows: the “Result and Discussion” section is divided into subsections relating to the individual measurement technique used, presenting measurement results and their analysis for undissolved and dissolved samples. In the “Conclusion” section, the most important conclusions are presented, and the “Experimental” section describes in detail the samples and measurement used.

## Results and Discussion

### Microstructure

**Figure 1:**
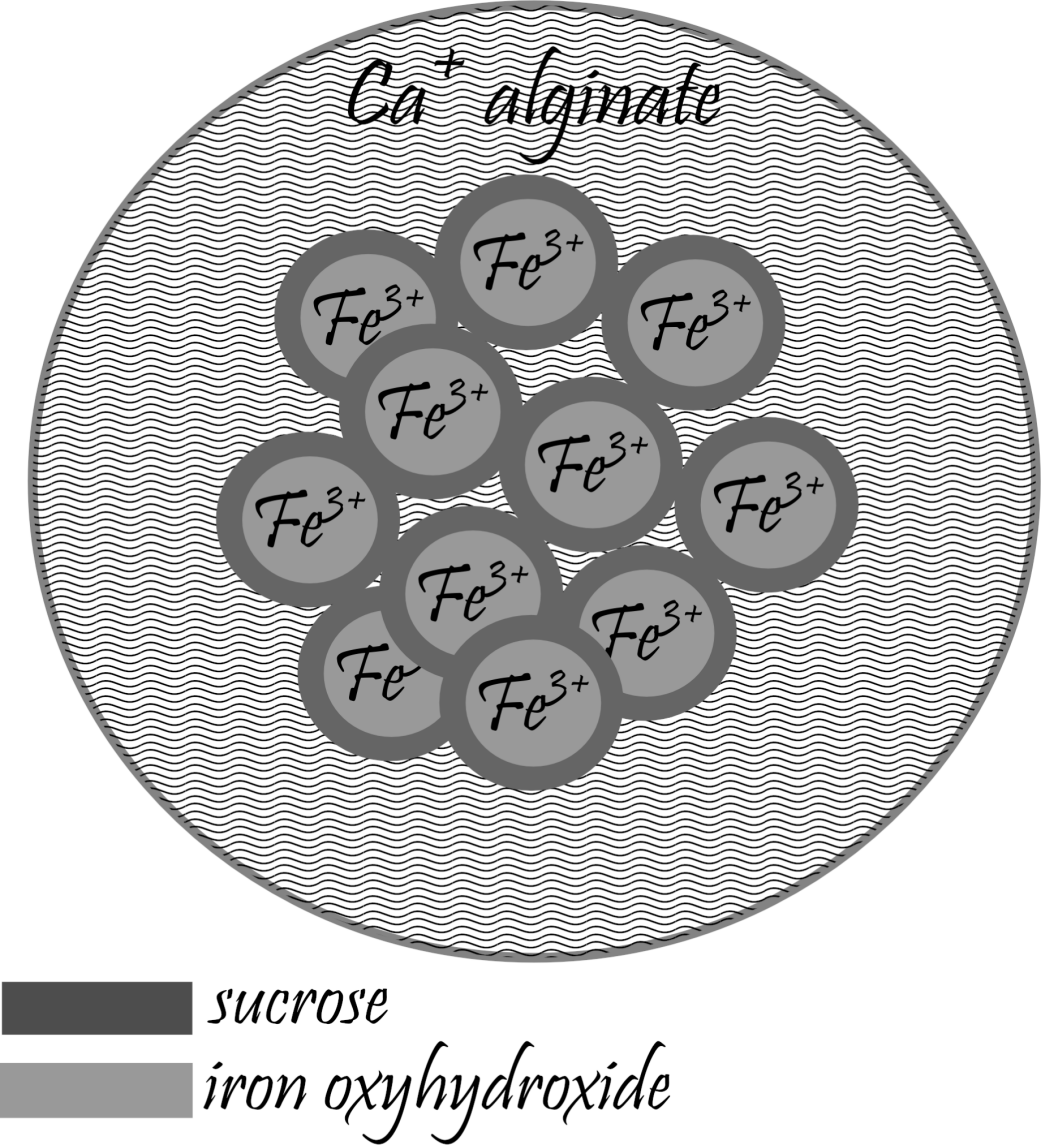
Model of the microcapsule studied.

Based on the studies and information published in [2,7–8,20], we created a hypothetical structure of microcapsules, which are the source of iron ions in the investigated samples (Figure 1). The assumption is that each individual microcapsule contains multiple core–shell nanoparticles where an iron oxyhydroxide core is covered by a sucrose molecule shell. This unknown quantity of core–shell nanoparticles is encapsulated within a Ca alginate coating.

**Figure 2:**
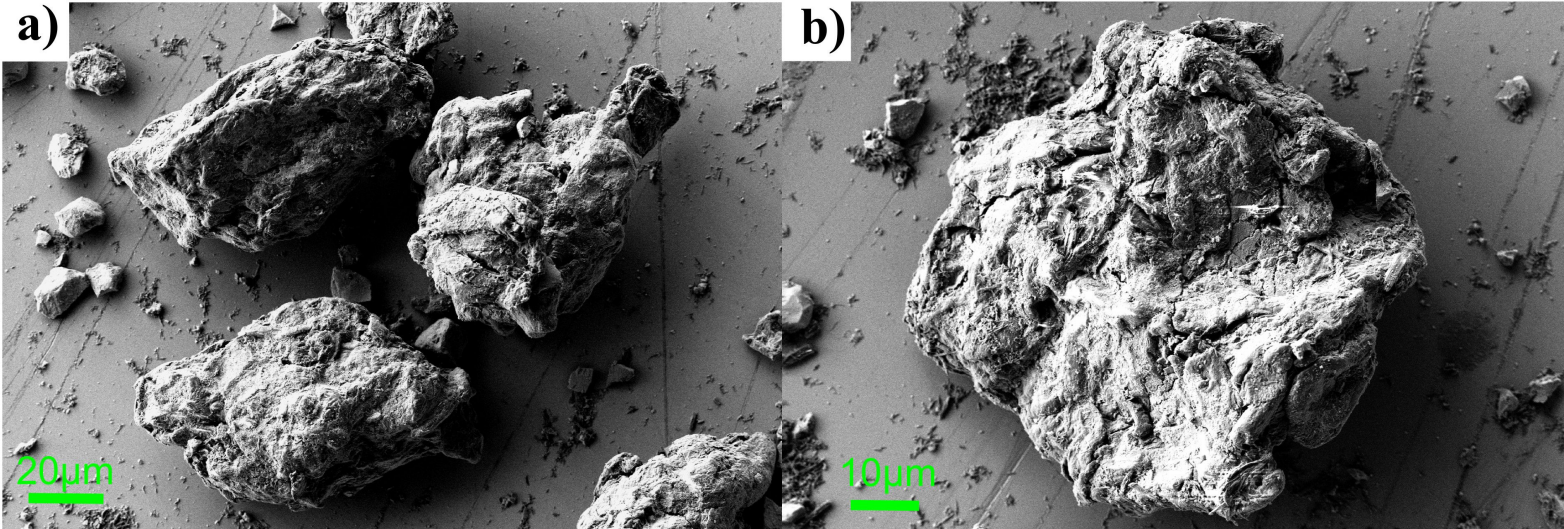
SEM images of the microcapsules from sample FS0. a) A set of three microcapsules with an elongated shape, and b) a single microcapsules with a regular shape.

SEM and TEM studies were conducted to compare the postulated structure of the microcapsule with its actual image. Figure 2 shows representative SEM micrographs of the FS0 sample. The observed grains exhibit various shapes and dimensions of several dozens of micrometers.

The surfaces of the grains are notably rough and torn, and during the measurement displayed high susceptibility to electron beam charging, which is a characteristic of organic compounds. Consequently, these grains were recognized as microcapsules. The resolution of the SEM technique is insufficient to recognize iron sucrose nanoparticles. Therefore, it is impossible to determine whether calcium alginate uniformly covers all iron sucrose nanoparticles or if some nanoparticles are on the microcapsule surface. Furthermore, it remains unclear if there is a fraction of iron sucrose that is not bound into microcapsules. Beyond the grains depicted in Figure 2, SEM measurements also registered elongated shreds and numerous smaller grains with more regular shapes and flat surfaces (not shown in the text). The former is most probably reflecting cellulose, while the latter is speculated to be vitamins and SiO_2_.

**Figure 3:**
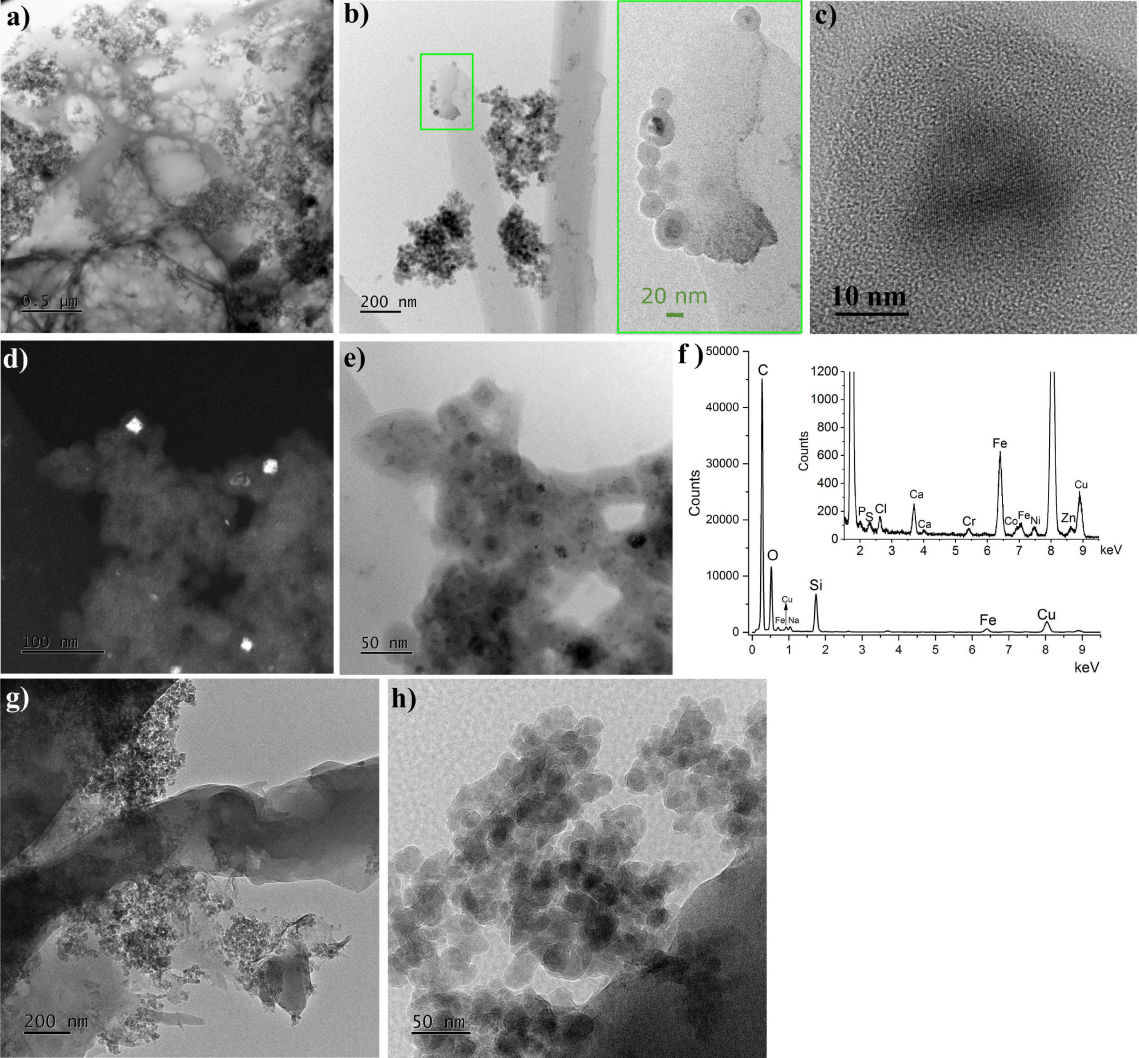
TEM images of the FS0 (a–e) and FST (g,h) samples. The green rectangle on the left shows the magnification of the selected area of (b). (f) Energy-dispersive X-ray spectroscopy results for the sample FS0.

Figure 3 illustrates TEM images of the samples FS0 and FST. For the FS0 sample (Figure 3a,b) groups of the nanoparticles are distributed in a porous structure (a) recognized as calcium alginate, but also attached to thin, elongated stripes probably representing cellulose (b). Figure 3b,e show that the core–sucrose shell structure of the nanoparticles in FS0 is clearly visible. Moreover, the dark-field image proved the presence of crystalline metallic cores (Figure 3d). It can be distinguished nanoparticles of larger, ≈18 nm, and smaller, <5 nm, cores, while the thickness of the shell seems constant ≈10 nm. The sucrose shell is homogenous; thus the individual cores appear to be well separated from each other. In Figure 3c, the high-resolution transmission electron microscopy (HRTEM) image presents a single crystalline nanoparticle. The lattice fringes are easily visible. The interplanar distance measured from the fast Fourier transform (FFT) is about 2.54 Å, which is in good accordance with the (400) plane of the α-FeOOH and with the (311) plane of the Fe_3_O_4_ phase, but less likely with the (40−1) or (400) plane of the β-FeOOH. Therefore, the question of the iron phase present in the FS0 sample appears to be unresolved based on the TEM results; however, the presence of the iron oxyhydroxide is highly probable. The energy-dispersive X-ray spectroscopy (EDS) spectrum (Figure 3f) of the FS0 sample collected from the area visible in Figure 3d confirms the presence, in addition to iron, of elements from additives and organic compounds, such as Si from SiO_2_, Ca from calcium alginate, and Co most likely from cobalamin. The Na and Cl peaks relating to salt (NaCl), and peaks of P, S, Ni, and Zn (trace levels), were classified as nonintentional dopants in the FS0 sample, while the Cu and Cr peaks are related to technical aspects of the measurement. TEM results of the FST sample (Figure 3g,h) state in contrast to the FS0 sample. Many irregular, nanoscale objects attached to micrometer ones can be distinguished, however the core–shell structure vanished. In the TEM dark-field images, the metallic cores were not stated, which suggests that observed nanoscale structures represent post-dissolution residues such as water-insoluble SiO_2_ or cellulose. The lack of core–shell structures in TEM images taken from FST does not indicate their total dissolution in physiological salt. TEM is a strongly selective technique in which only a tiny sample volume is analyzed; however – as shown further in the text – the TEM results are consistent with the magnetic measurements.

### XRD results and analysis

**Figure 4:**
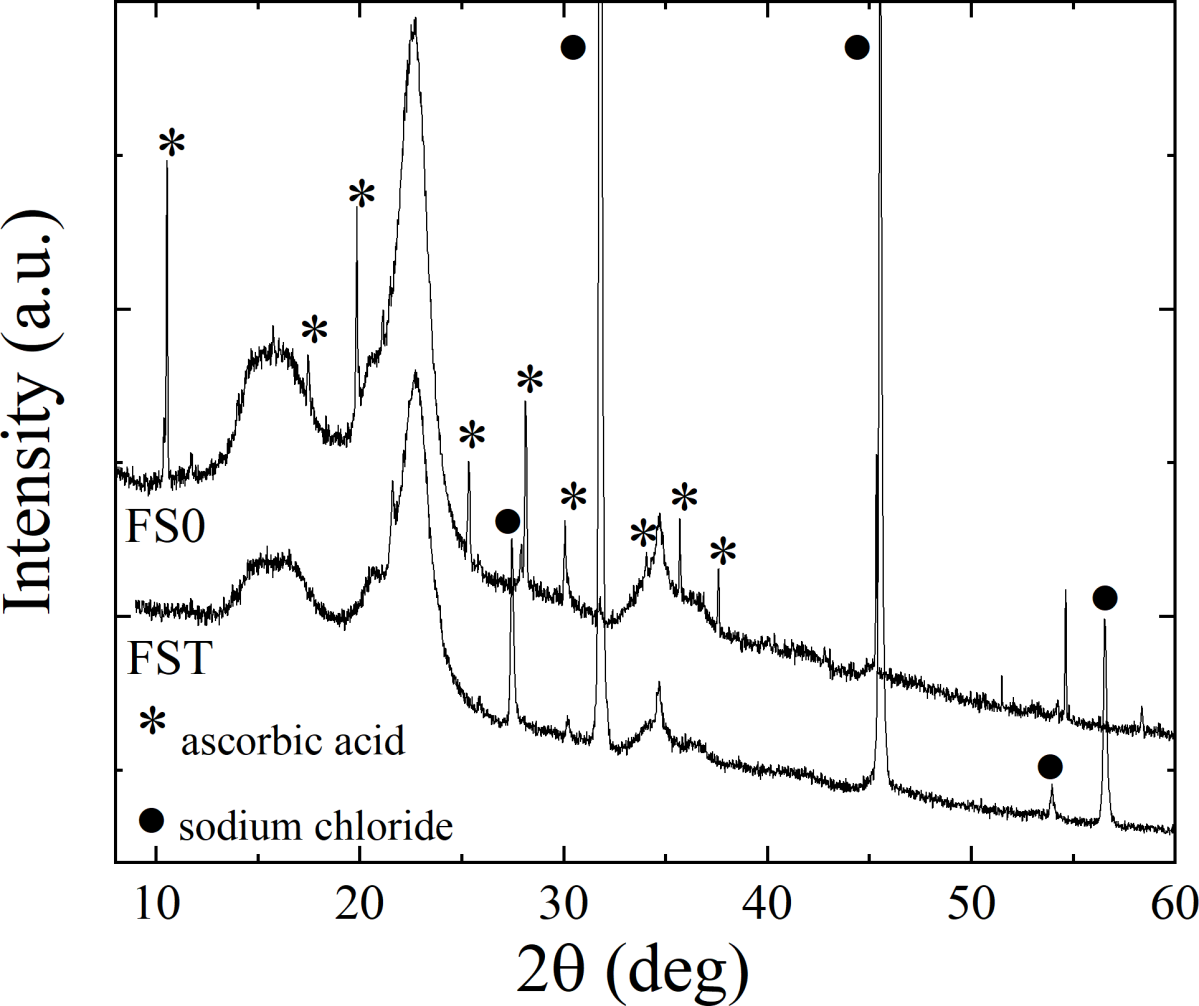
X-ray diffraction patterns of the FS0 and FST samples.

Figure 4 shows the XRD patterns of the FS0 and FST samples. In both patterns, three broad features can be distinguished with the same localization of maxima around 15.6°, 23°, and 35°, and an additional arrangement of sharp lines at different angular positions. These three dominant lines in both XRD patterns most probably belong to amorphous microcellulose as the obtained pattern agrees well with the literature [21–22]. There are no changes in these lines between both samples which is associated to the fact that cellulose interacts with water but is insoluble in it. It was expected that lines related to SiO_2_ [23–24] for both samples, and to sucrose [4,6,8,14] and calcium alginate [25–26] for the FS0 sample would be registered in the XRD pattern. However, they were most likely covered by the cellulose peaks due to the similarity of angular positions.

For the FS0 sample, the sharp peaks appear at 10.46°, 17.46°, 19.82°, 25.36°, 28.03°, 30.11°, 34.03°, 35.65°, and 37.63° (marked with asterisks in Figure 4). According to [27–28], they reflect ascorbic acid present in this sample. The lack of these peaks in the pattern of the FST sample is related to the solubility of ascorbic acid in saline. The diffraction peaks (dot marks) in Figure 4 correspond to sodium chloride [29]. Their intensity significantly increases after the dissolution procedure, which indicates the crystallization of NaCl from saline.

The identification of the iron-containing phase based on the XRD data for the FS0 sample is problematic, as the most intense peaks predicted for the possible phase (i.e., any of the FeOOH polymorphs) are not observed. The XRD results also do not indicate the presence of iron oxides. The explanation to this is most probably a combination of two facts. The major contribution to the sample mass originates from the additives (ca. 95%), where cellulose dominates. Thus, in the same way for SiO_2_, sucrose, and calcium alginate, the possible Bragg peaks from the iron-containing phase are hidden under the background and cellulose signal. Another cause may be an amorphous structure of the nanoparticles, as it is well known that XRD patterns of nanoscale amorphous grains exhibit averaged, broad X-ray scattering maxima, in contrast to the sharp diffraction maxima from a crystalline material [30–31]. The prediction of the amorphous iron-containing phase is based on XRD contrasts with the TEM images, which registered the crystalline structure of the Fe-containing nanoparticles in the FS0 sample. However, the detection of isolated crystalline nanoparticles with an Fe-containing core does not exclude the possibility of the presence of other forms of iron-containing compounds in the FS0 sample. Comparing the XRD and TEM results for the FST sample, there is no doubt that they are consistent with each other, as in both cases there are no signs of Fe-containing phases.

### Magnetic studies (dc and ac) results and analysis

#### Undissolved sample FS0

**Figure 5:**
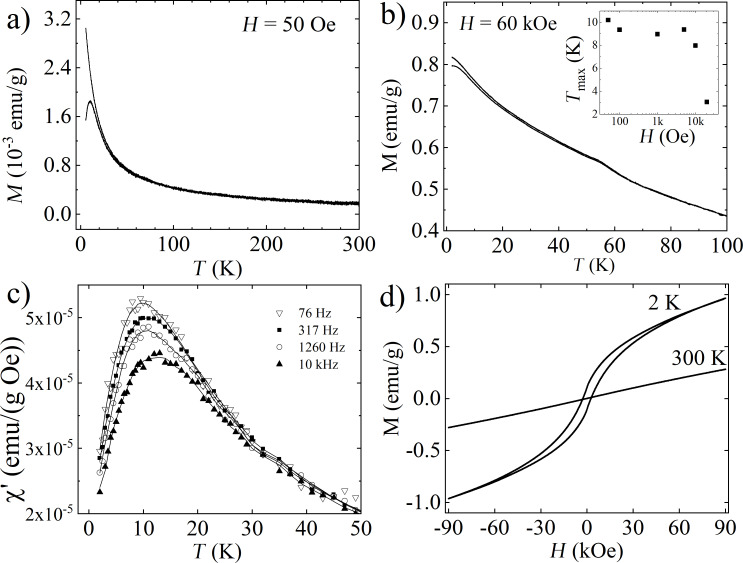
For FS0: ZFC–FC dependences registered at 50 Oe (a), and at 60 kOe (b). The inset in part (b) represents the magnetic field dependence of the *T*_MAX_ temperature. (c) The real part of the magnetic susceptibility as a function of temperature measured for several ac magnetic field frequencies – the relation was smoothed by a polynomial function (straight lines). (d) Magnetization curves registered at 300 and 2 K.

The results of static and dynamic temperature magnetic measurements of the FS0 sample are presented in Figure 5. Figure 5a presents the temperature dependences of magnetization recorded at 50 Oe. It shows that zero-field-cooled (ZFC) and field-cooled (FC) curves are indistinguishable above 60 K and behave significantly differently below this temperature. As the temperature decreases, the value of the magnetization of the curves slightly increases, then a low temperature maximum, at *T*_MAX_ ≈ 10 K, appears in the ZFC curve, while the magnetization value of the FC curve begins to more rapidly increase. The observed maximum in the ZFC relation is sharp, and this is the only singularity visible in this curve in the considered temperature range.

Increasing the magnetic field to 10 kOe did not significantly change the position of *T*_MAX_ (see inset of Figure 5b), and the shape of the ZFC–FC relation. Only the magnetic field above 10 kOe shifts the ZFC maximum to lower temperatures; however, even at 60 kOe, the full reversibility of the ZFC and FC curves is not achieved (Figure 5b). The so-called irreversibility temperature, *T*_IRR_, at which the ZFC and FC curves bifurcate, is very little dependent on the magnetic field and is around 65 K for all registered temperature relations. It should be also stressed that in the *M*(*T*) relations there is no trace of the Verway transition (*T*_V_ ≈ 125 K) typical for Fe_3_O_4_. Figure 5c presents the temperature dependences of the real part of the magnetic susceptibility, χ’(*T*), registered at several frequencies *f* of an ac external magnetic field for FS0. (In Figure 5c only chosen relations are presented, while the ac magnetization studies have been also performed for other values of *f*.) In the χ’(*T*) relation for the FS0 sample, a peak with the maximum in the vicinity of *T*_MAX_ is observed, whose location changes with frequency from 9.8 K for *f* = 40 Hz to 13 K for *f* =10 kHz. It should be added that due to the relatively low signal from the FS0 sample, the recorded χ’’(*T*) curves (not shown here), made it impossible to determine any peak or other anomalies. Figure 5d presents the magnetization curves recorded for the FS0 sample at two extreme temperatures, 300 and 2 K. (The *M*(*H*) curves recorded between these temperatures were not plotted. Only the temperature dependences of the coercivity and the remanence field are presented further in the text). At 300 K, the magnetization almost linearly increases with the magnetic field and the curve is reversible, while at 2 K the *M*(*H*) curve exhibits a non-zero coercivity of ≈2630 Oe, but it neither saturates nor overlaps in the field range used.

From the pharmaceutical point of view, the shell is used to slow the release of the iron after the supplementation, and also to stabilized the magnetic core. Whereas, by treating the microcapsule as a magnetic system, it is expected that the sucrose shell will prevent or significantly diminish interactions between nanoparticles, also probably modifies the surface state of the core.

**Figure 6:**
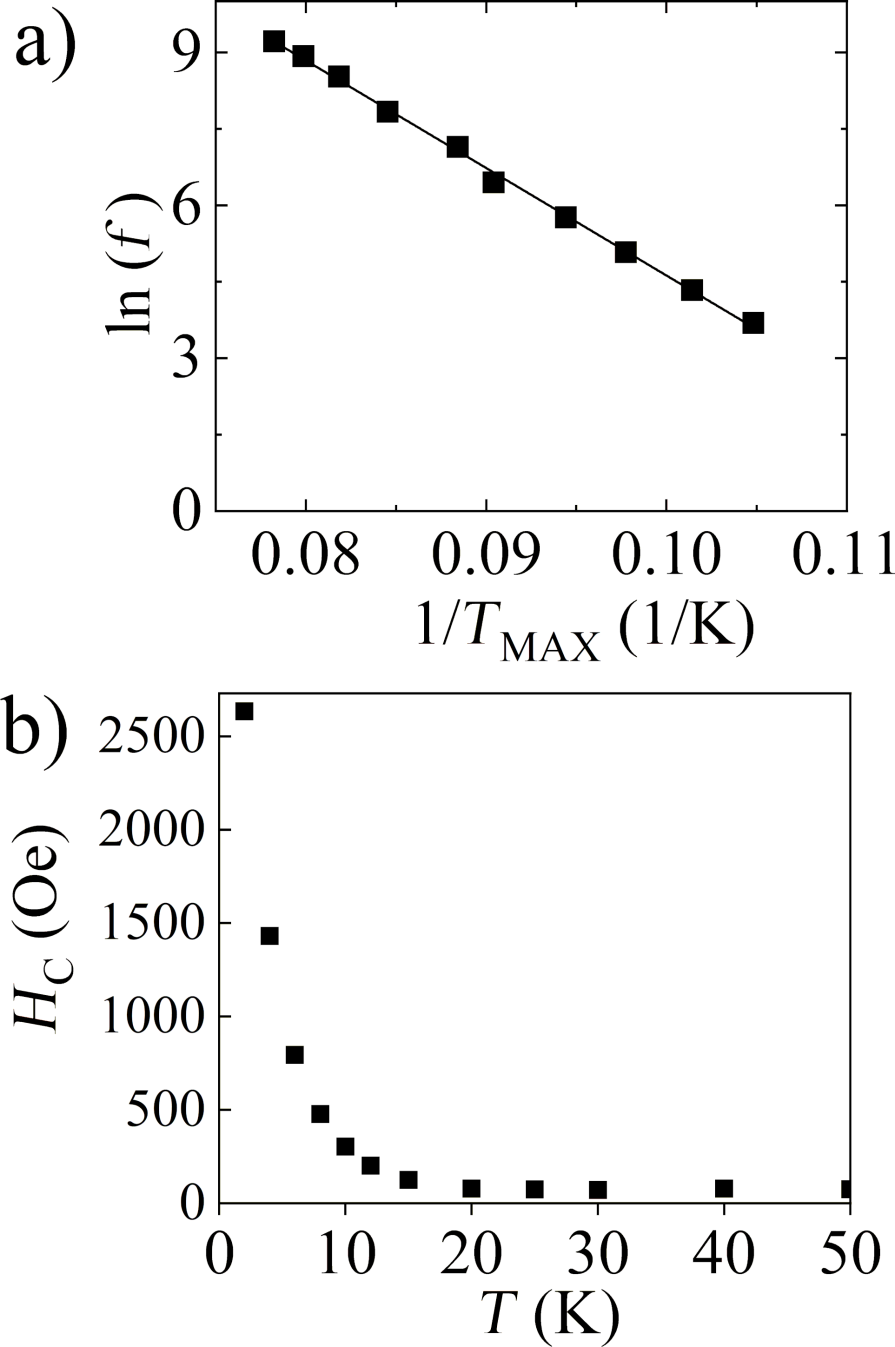
For FS0: (a) Dependence of the ac magnetic field frequency with the inverse *T*_MAX_ temperature (points). The fitted relaxation model for superparamagnetic nanoparticles is represented by the straight line. (b) Thermal dependence of the coercivity.

To understand the low-temperature magnetic state of the FS0 sample, the zero field cooled – field cooled relations (Figure 5a) will be firstly analyzed. Recording the low temperature maximum observed in the ZFC curve, together with the sharp increase of the FC curve with the temperature decrease, most likely visualizes the blocking process of superparamagnetic nanoparticles with the temperature decrease. The first step in the verification, whether the blocking process of individual nanoparticles is observed, was the analysis of the ac magnetic measurement results. The dependence between the temperatures of the χ’(*T*) maxima, *Τ*_ΜΑΧ_, and frequency of the ac magnetic field was specified. To the gathered data plotted as lnτ(*Τ*_ΜΑΧ_^−1^) dependence, the below relaxation model was fitted [32]:


[1] $$\tau =\tau_{0}\exp\left(\frac{E_{A}}{k_{\text{B}}T_{\text{MAX}}}\right),$$

where τ = *f*^−1^, *k*_B_ is the Boltzmann constant, τ_0_ is the characteristic relaxation time, and *E*_A_ is the activation energy of a nanoparticle. The fit gave the value τ_0_ ≈ 6.8 × 10^−12^ s, which is in the predicted range (i.e., 10^−9^–10^−14^) for the blocking process of single particles, and *E*_A_/*k*_B_ ≈ 211 K, which is also reasonable [33]. The results of the fit are visualized in Figure 6a.

Additional verification of the connection of the maximum registered in the dc and ac temperature magnetic measurements for FS0 with the blocking process of the noninteracting nanoparticles was carried out by the calculation of the empirical parameter Φ = Δ*T*_MAX_/(*T*_MAX_·Δlog_10_(*f*)), which describes the shift of the *Τ*_ΜΑΧ_ with the frequency [34]. The obtained value of Φ = 0.14 corresponds to the literature values characteristic for the blocking process of superparamagnetic nanoparticles [35]. This means that the iron compound in the studied sample has a form of noninteracting nanoparticles; thus, the proposed model of microcapsules seems to be correct. Also, the coercivity presented in Figure 6b, which significantly increases with temperature lowering, very well reflects the blocking process in the sample.

Having the confirmation of the blocking process in the sample, the modified Langevin function was fitted to the magnetization curves collected in the temperature range from 70 to 250 K, where the sample should exhibit superparamagnetic behavior. The *M*(*H*) relation registered at 300 K was not included in the fitting due to the linear character. It should be highlighted that, although the iron sucrose is only 5% of the Ferisan mass, the nonmagnetic contribution from the additives, which can be expected especially in the *M*(*H*) curves registered at higher temperatures, is not visible even at high magnetic fields. This indicates the predominance of the signal from iron sucrose in comparison to other sample components. Another conclusion from the non-saturated shape of the *M*(*H*) relations registered for the FS0 sample regardless of the measurement temperature is that such behavior is typical for antiferromagnetic systems. This conclusion correlates with the TEM results which predicts the antiferromagnetic α-FeOOH or, with a lower probability, the β polymorph in the nanoparticle cores.

**Figure 7:**
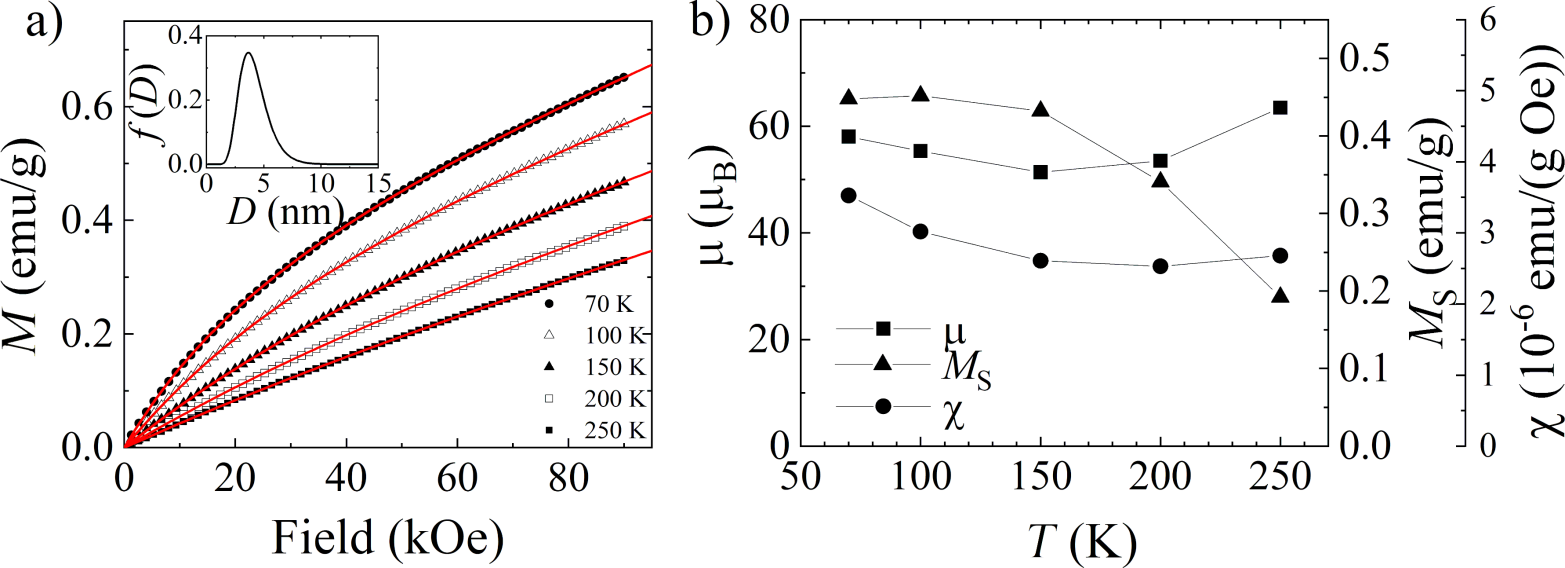
For the FS0 sample: a) *M*(*H*) dependences (points with the fitted modified Langevin function (line)). The inset presents the obtained log-normal distribution of nanoparticle sizes, where <*D*> = 4 nm and σ = 0.3. b) μ, *M*_S_, and χ obtained by the fitting Equation 2 to the *M*(*H*) dependences.

The fitting equation used was as follows:


[2] $$M=M_{\text{S}}\int_{0}^{\infty }L\left(\frac{\mu B}{k_{\text{B}}T}\right)\cdot f\left(D\right)\text{d}D+\chi \cdot B,$$

where μ is the magnetic moment of a single nanoparticle and depends on its dimension, *f*(*D*) is a log-normal distribution of the nanoparticle diameters and it was the same in each fit, and *M*_S_ is the saturation magnetization of the sample [32]. The χ·*B* term in Equation 2 describes the total linear component of the magnetization curves which expresses the possible antiferromagnetic susceptibility of the nanoparticles [35–37], and paramagnetic and diamagnetic contributions from the additives and from the sample holder (if any). It should be noted that μ, *f*(*D*), *M*_S_, and χ were the fitting parameters. One of the start values in the fitting procedure was the median of *D*, named <*D*>, and its standard deviation σ, which were difficult to estimate as the distribution of nanoparticle diameters *f*(*D*) was not known. Following the literature values for iron sucrose nanoparticles in other hematinics [2,7–8,13], and the temperature position of the ZFC peak, the range between 2 and 7 nm was chosen. After the first round of fitting, <*D*> was estimated to be 4 nm, and σ to be 0.3 nm. The results obtained for other <*D*> and σ values, even though there was good agreement between measurement points and fitted curves, were unphysical in different ways. For example, in the fits where <*D*> was equal to 7 nm the increase of μ with the temperature was registered, which has already been described in [36] for the antiferromagnetic nanoparticle system, as a dependence without physical ground. The next round of the fitting procedure was done for fixed <*D*> and σ values, which allowed obtaining the remaining fitting parameters. In Figure 7a, which presents the *M*(*H*) relations for the FS0 sample at 70, 100, 150, 200, and 250 K, the lines present the fitted model to the experimental data for <*D*> = 4 nm and σ = 0.3, and the inset shows the obtained particle size distribution. It is well visible that the predicted model describes the experimental data perfectly. The small <*D*> value of the nanoparticles found in the fitting, and a quite narrow *f*(*D*) distribution coincide with the shape of the ZFC curve for the FS0 sample (Figure 5a), and with the TEM images (Figure 3a–e). For a noninteracting system of magnetic nanoparticles, the temperature at which the blocking process begins directly depends on the mean particle size and its distribution in the sample. Here, the narrow maximum in the ZFC curve located at low temperatures signaled the small particle size just after the measurement, and the calculation confirmed this assumption.

Figure 7b presents the temperature dependence of the parameters *M*_S_, μ, and χ obtained in the fitting. It should be stressed here that obtained values of the fitting parameters together with the shape of the *M*(*H*) relations do not indicate that the major phase of the nanoparticle cores can be ferromagnetic Fe_3_O_4_. Conversely, the low level of calculated *M*_S_ and μ values stays in line with the thesis of the antiferromagnetic arrangement of the magnetic moments in nanoparticle cores, as for an antiferromagnetic nanoparticle only uncompensated spins transform into the net magnetic moment. In this context, the presence of FeOOH in the cores seems to be the most possible hypothesis.

The μ value was estimated within the range ≈50–65 μ_B_ and exhibits only slight changes with temperature. Assuming that typically the magnetic moment of Fe^3+^ ions in the iron oxyhydroxides is in the range of ≈3–4 μ_B_ [38–40], a number of uncompensated spins, named *n*_UC_, in the nanoparticle core does not exceed 20. Such extremely low values of μ and *n*_UC_ were the reason for comparing them with the models proposed by Néel [41] for an antiferromagnetic nanoparticle consisting of *n*-spins. The first model states that the number of uncompensated spins equals to 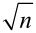 for randomly distributed spins in a volume of a nanoparticle, while the second model predicts the number of surface uncompensated spins equals to 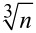. Assuming that the volume of the 4 nm diameter nanoparticle is ≈351 × 10^−21^ cm^3^, and the average number of Fe^3+^ ion per cm^3^ for all FeO(OH) polymorphs is ≈2.5 × 20^22^ Fe^3+^·cm^−3^ [16], the obtained values were as follows: *n* ≈ 880, 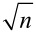 ≈ 30, to 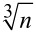 ≈ 10. The quantities of the uncompensated spins calculated from the Néel models are at the level similar to that obtained in the fitting of the modified Langevin function to the *M*(*H*) dependences for the FS0 sample. It means that the assumptions about the core composition from oxyhydroxide seems to be accurate. The estimated *n*_UC_ value falls within the range of calculated 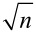 and 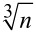, which would mean that the uncompensated spins are located not only at the surface of the cores. It is known that the chemical bonding between the sucrose and the iron oxide surface ions is very likely to reduce the surface net magnetic moment, as the missing bonds on the surface may be replaced by the sucrose ligands. Such a situation may restore the antiferromagnetic arrangement within the surface layer, and consequently reduce the quantity of uncompensated spins in the sample [4,19]. In this context, it is more likely that the source of the net magnetic moment is the interior volume of the core than of its surface layer. It should be added here that the presence of the uncompensated spins randomly distributed within the nanoparticle core volume, indicating poor crystallinity, is in line with the absence of high intensity, sharp peaks from the Fe phase in the XRD spectrum.

Small values of the nanoparticle magnetic moment and saturation magnetization for iron oxyhydroxides are often reported [5,42–43]. However, the direct comparison of μ and *M*_S_ values obtained here with those from the literature may not perfectly coincide due to differences in shape, dimensions, quality of the crystalline structure, and the presence of a nonmagnetic coating or matrix, as all this structural features have a strong impact on the magnetic properties of the nanoparticle system [40,42,44].

The analysis carried out confirms the presence of noninteracting magnetic nanoparticles in the FS0 sample and proved that most likely the cores of the nanoparticles are composed of FeO(OH). However, the results of the magnetic measurements do not allow resolving the issues related to the type of FeO(OH) polymorph. Therefore, additional studies such as FTIR, Raman, and XPS were performed, and their results will be presented later in the FTIR and Raman results and analysis and XPS results and analysis sections.

#### Dissolved samples

**Figure 8:**
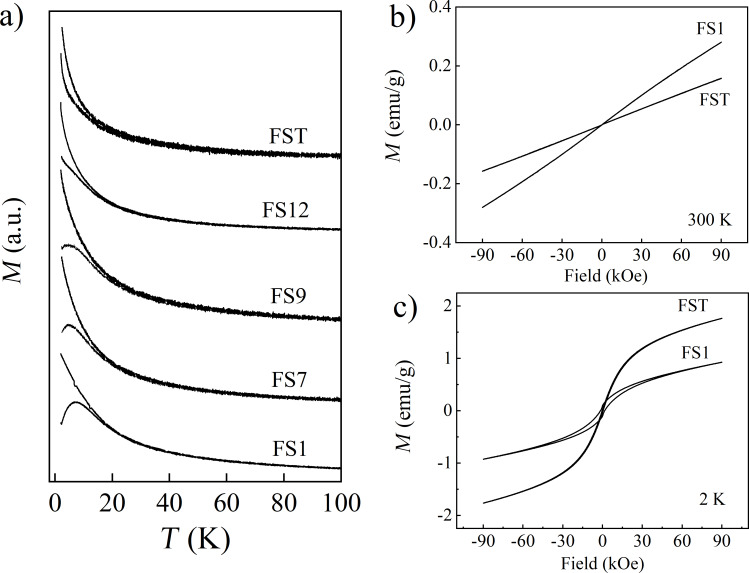
(a) Temperature dependences of the magnetization measured at 50 Oe in the ZFC–FC modes for selected samples FS0, FS1, FS7, FS12, and FST (shifted by the constant values along the *y*-axis). (b), (c) Magnetization curves registered at 300 and 2 K respectively, for the selected two samples FS1 and FST.

The evolution of the magnetic properties of FS0 with the dissolution time is shown in Figure 8. Figure 8a depicts ZFC–FC relations measured at 50 Oe for all our samples. It can be seen that as the dissolution time increases, the ZFC maximum shifts to lower temperatures and then decays for times *t* ≥ 12 h (the values of the *T*_MAX_ temperatures are listed in Table 1). It needs to be highlighted that for the FST sample (i.e., infinite time of dissolution) the irreversibility of the ZFC–FC curves is still well defined. All *M*(*H*) dependences recorded at 300 K for dissolved samples are linear with the magnetic field (exemplar curves in Figure 8b). The magnetic susceptibility values calculated for *H* = 10 kOe are gathered in Table 1, and are not a monotonic function of the time *t* (i.e., the value obtained for the FST sample is placed between the values for FS1 and FS7 samples). From the *M*(*H*) relations collected at 2 K (exemplar curves in Figure 8c) for the dissolved samples, it can be concluded that the coercivity values decrease with increasing dissolution time (see Table 1). It should be noted for the FST sample that *H*_C_ is well defined and has value of 285 Oe.

**Table 1:** *T*_MAX_ temperature (i.e., the temperature of the ZFC curve maximum), values of the magnetic susceptibility χ at 10 kOe and 300 K, and *H*_C_ values at 2 K for all samples investigated.

	*T*_MAX_ (K)	χ (emu·g^−1^·Oe^−1^) 300 K, 10 kOe	*H*_C_ (Oe) 2 K

FS0	10.3	3.40 × 10^−6^	2630
FS1	7.2	2.88 × 10^−6^	1560
FS7	4.8	1.40 × 10^−6^	1225
FS9	4.3	1.13 × 10^−6^	1145
FS12	–	1.12 × 10^−6^	646
FST	–	1.86 × 10^−6^	285

In order to understand the FS0 sample dissolution process and connect it with the changes of the magnetic properties presented in Figure 8, the following analysis was performed. Based on it and on the knowledge about the investigated compound, we assumed that the calcium alginate microcapsules are embedded in the additive matrix; however, it does not interact with the matrix. Therefore, the additives do not disturb in any way the microcapsule dissolution process, and the interaction between saline and microcapsules happens immediately after their connection.

**Figure 9:**
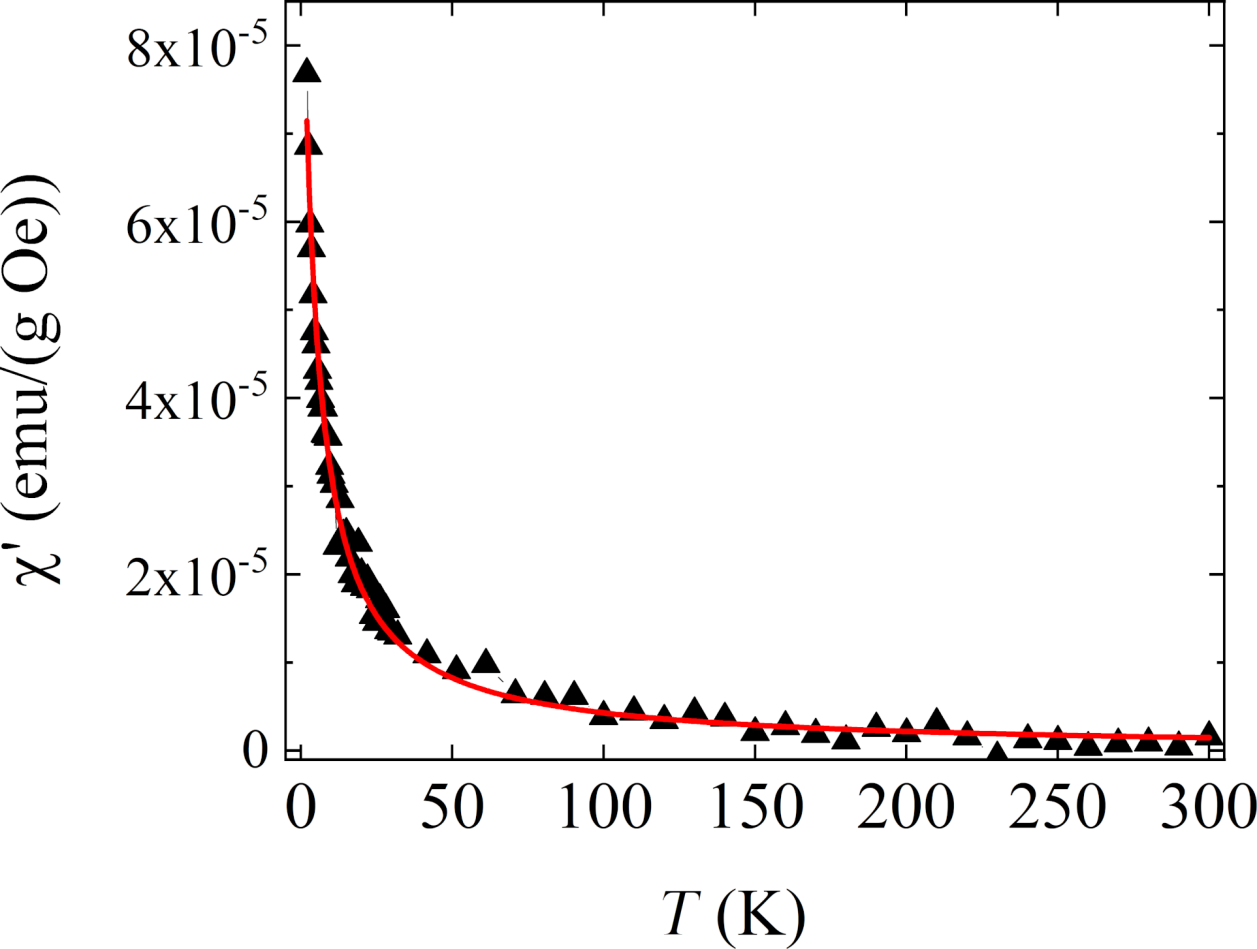
The real part of the magnetic susceptibility as a function of temperature measured for FST at *f* = 1.25 kHz (black triangles) with fitted modified Curie–Weiss law (red line) curve. Parameters obtained in the fit: *C* = 4.47 (emu∙K)/(g∙Oe), θ = −4.3 K, χ_0_ = 0.

The ZFC–FC dependences registered for the dissolved samples, plotted in Figure 8a, are a reflection of the structural changes which take place in the FS0 sample with the dissolution time. Intuitively, in the outermost layer the calcium alginate of the microcapsule should dissolve first. (The dissolution of the calcium alginate was proved by the XPS studies, and it will be presented and comment in section XPS results and analysis.) The next step is the dissolution of sucrose; whose molecules are polar, thus, they are not a barrier for saline. Without the sucrose shell, the iron oxyhydroxide core being in direct contact with saline may undergo through decomposition/degradation process. From the superparamagnetism theory, the maximum temperature observed in the ZFC dependence is closely linked to the mean value of the particle diameter <*D*> in the grain nanosystem [45], and its shifts to lower temperatures with a decrease in <*D*>. Therefore, for the samples with the dissolution times < 12 h, the observed shift of the ZFC maximum to lower temperatures may be associated with the beginning of the decomposition process of the iron oxyhydroxide nanoparticles in contact with saline [46]. The disappearance of the ZFC peak for the FS12 and FST samples also fits into these assumptions, and supposedly means the decrease of the core diameter to the ultra-low value for which the blocking process vanish. The result obtained for FST well coincide with the TEM images where metallic cores were not registered. It should be added that the sharp decrease of *H*_C_ with the dissolution time, at 2 K and 2500 Oe for the FS0 sample to 285 Oe for FST, also well reproduces the idea of nanoparticle decomposition with dissolution time. However, the well-defined irreversibility region of the ZFC and FC curves for the FS12 and FST samples, as same as nonzero coercivity, indicates that these samples are not purely paramagnetic. This means that, as a result of dissolution in the physiological saline, there was no complete decomposition of the iron oxyhydroxide core into paramagnetic iron ions. It may indicate the presence of some exchange coupled iron ions in the FST sample. The type of magnetic interactions between remaining Fe ions are hard to determine, since most probably the Fe ions are randomly dispersed in the polymer residues, which translates into random distribution of bond lengths and orientations. However, one attempt of the classification of the magnetic interactions in the FST sample was the description of the χ’(*T*) dependence with the modified Curie–Weiss law: 
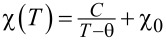
; where *C* is the Curie constant, χ_0_ is the susceptibility component independent of the temperature, and θ is the parameter often called the Curie–Weiss temperature, whose sign indicates the type of magnetic interactions in the investigated system (the results are shown in Figure 9).

It was found here that θ = −4.3 K, indicating the dominance of the antiferromagnetic exchange interactions in the FST sample between the Fe ions, which stay coupled after the dissolution procedure. It should also be added that the rapid increase of χ’ in the low temperature region indicates the existence of a paramagnetic contribution in the FST sample. This means that in the FST sample a coexistence of the antiferromagnetic and paramagnetic contributions become from interacting and noninteracting Fe ions, respectively, can be distinguished.

Due to the complex magnetic nature of the FST sample, it was tested using FTIR, Raman, and EPR methods. More information about its magnetic state will be reported in sections FTIR and Raman results and analysis and EPR results and analysis.

#### FTIR and Raman results and analysis

**Figure 10:**
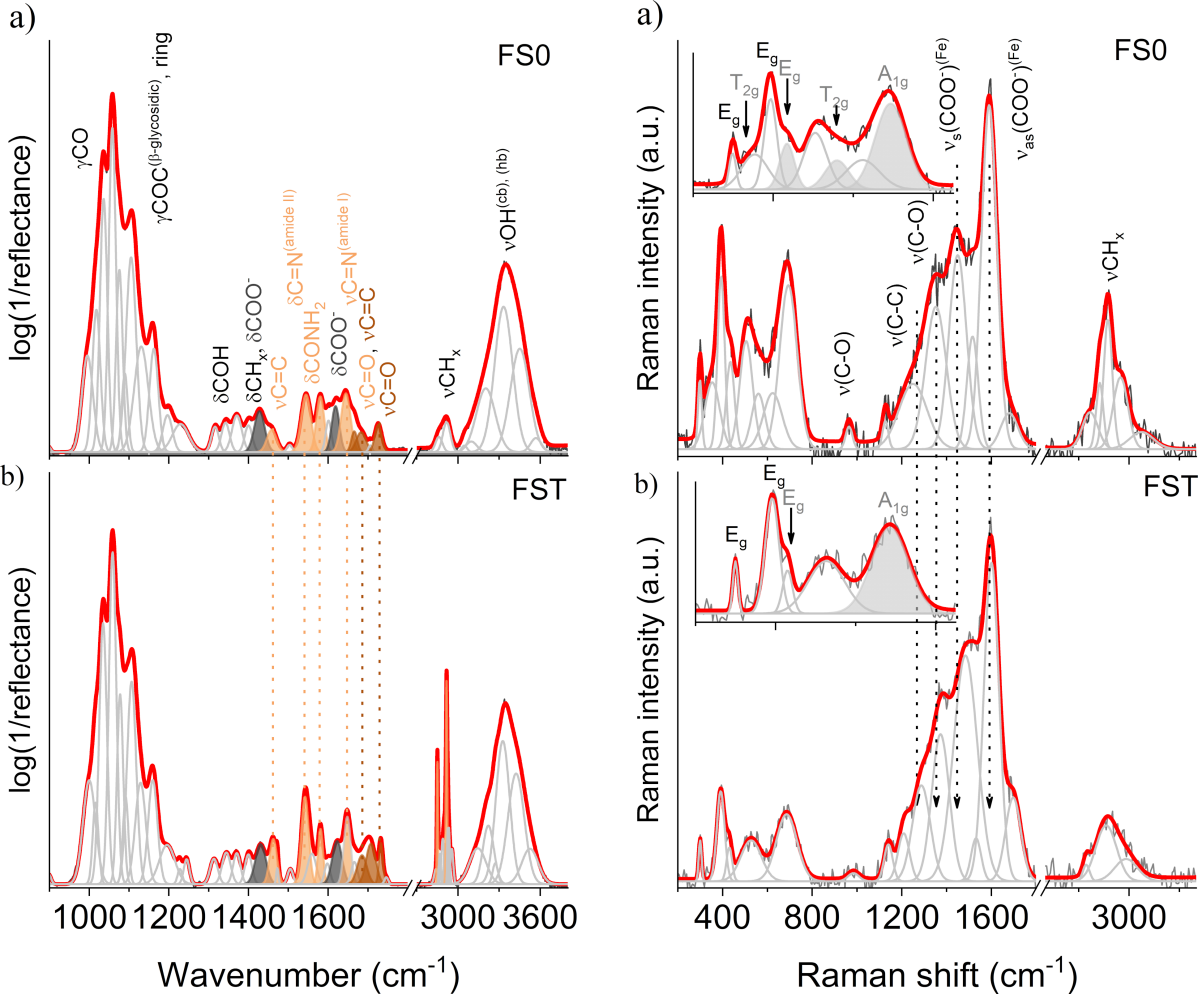
Infrared (left panels) and Raman spectra (right panels) of FS0 (a) and FST (b) summarized in the regions 900–3800 cm^−1^ (FTIR) as well as 200–3200 cm^−1^ and 200–850 cm^−1^ (Raman). The signal originated from individual components (FTIR: yellow – vitamin, dark grey – calcium alginate; Raman spectra: light grey – magnetite), considering characteristic types of bonds or symmetry vibrations were highlighted in color. FTIR and Raman spectra were fitted using the Voigt function to separate individual organic and inorganic phases.

FTIR and Raman spectroscopy techniques were employed to investigate the phase composition and iron oxide type resulting from the synthesis and to validate structural alterations after interaction with saline (see Figure 10).

The FTIR spectra of FS0 and FST are predominantly characterized by bands related to polysaccharides due to their structural similarities with cellulose, calcium alginate, and sucrose [47]. Consequently, many of these bands overlap with each other. The most prominent bands are observed within the range of 3600–3200 cm^−1^, with weaker bands in the vicinity of 3000–2800 cm^−1^. The former bands originate from stretching vibrations of hydroxyl groups, encompassing both covalent (cb) and hydrogen bonds (hb), as well as aliphatic CH*_x_* groups, where *x* = 1,2 [48–54].

The infrared spectra of FS0 and FST provide meaningful insights into their structural characteristics, with a high sensitivity to subtle changes in their composition. These spectra prominently highlight the distinctive cellulose-band arrangement, featuring well-defined bands associated with internal deformation vibrations of CH_2_ within CH_2_OH groups at approximately 1430 cm^−1^ [47]. The deformation vibrations of C–OH and CH groups were within the range of 1400–1300 cm^−1^ [55], as well as the stretching vibrations of C–O–C within the β-glycosidic ring, external deformation vibrations of CH_2_ groups, and the C–C and C–O modes within the β-D-glucose ring, within the range of 1200–1000 cm^−1^ [56].

The infrared spectra of FS0 and FST also reveal calcium alginate-related bands attributed to deformational modes of carboxylate COO^−^ ions, resulting in asymmetric and symmetric stretching C=O vibrations at 1620 and 1430 cm^−1^ [51,53]. However, a remarkable similarity of functional groups of different considered polysaccharides leads to the overlapping of many bands, making it challenging to unequivocally distinguish other calcium alginate or sucrose-related features [51,54].

The most notable distinctions between the FS0 and FST spectra become evident in two key regions: 3000–2800 cm^−1^ and 1750–1400 cm^−1^ (see Figure 10). In the former, the bands related to methyl and methylene groups overlap with the more intense polysaccharide features in the FTIR spectrum of FS0. On the other hand, the infrared spectrum of FST stands out with sharp and intense bands at 2919 and 2837 cm^−1^, primarily derived from methylene CH_2_ groups. In the latter region, bands associated with the vibrational modes of functional groups in the vitamin molecular backbone, potentially vitamin C (ascorbic acid) and/or vitamin B9 (folic acid), exhibit slight variations depending on the system formulation. Thus, the FS0 and FST infrared spectra in the fingerprint region revealed ascorbic acid-related bands at approximately 1725/1734 and 1673/1683 cm^−1^ (FS0/FST) attributed to the C=O stretching and five-membered lactone ring modes, coupled with neighboring vibrations within the conjugated system of the molecule [57–59]. In turn, distinct bands on the FS0 spectrum at 1669, 1645, 1580, 1542, and 1462 cm^−1^ derived from the stretching vibrations of –C=O groups within the pterin structure, –C=N stretching (amide I), –CONH_2_ bending [60], –C=N stretching (amide II), and –C=C stretching of phenyl and pterin rings [61] of folic acid. Of note, the infrared spectrum of FST displays an increased intensity in the folic acid-related bands, a slight upshift in the ascorbic-related bands, and the emergence of more pronounced CH_2_ bands. This behavior suggests potential conformational alterations or molecular reorganization of the vitamin structure due to forming metal (calcium/sodium/iron) complexes. The changes in the vitamin structure observed here are consistent with the XRD results, in which the peaks related to ascorbic acid are observed in the spectrum of the FS0 sample, while they vanished in the FST spectrum.

Raman spectroscopy confirmed the formation of an iron-stabilized biopolymer ionic cross-linking complex. This confirmation was based on an analysis of intensity, full width at half maximum, and band shifts. In general, both FS0 and FST Raman spectra exhibited a similar pattern. They included a complex of bands in the 3000–2800 cm^−1^ region related to CH*_x_* modes and strong bands in the 1600–1000 cm^−1^ region. These bands originated from carboxylate and glycosidic modes, which are structurally sensitive to interactions with metal surfaces and the formation of organometallic complexes [62–64].

A closer examination of FS0 and FST spectra reveals some distinctions (Figure 10). The Raman spectrum of FS0 contained a higher number of CH*_x_* bands with higher intensity compared to the spectrum of FST. Furthermore, in the fingerprint region, FS0-related bands associated with COO^−^ stretching modes were separated into asymmetric (1588 cm^−1^) and symmetric (1451 cm^−1^) stretching, C–O stretching vibration (1345 cm^−1^), and glycosidic ring breathing mode (1133 cm^−1^) [65]. After immersion in physiological salt (NaCl) solution, these bands were upshifted while the full width at half maximum simultaneously increased by approx. 5 cm^−1^ (Figure 10). Conversely, the Raman spectrum of FST featured asymmetric and symmetric COO^−^-related bands (1598, 1480 cm^−1^), C–O stretching vibration (1363 cm^−1^), and glycosidic ring breathing mode (1196 cm^−1^) [65].

One possible explanation for the observed changes in both regions could be a structural reorganization or a conformational effect of the polysaccharide molecules, possibly driven by the structural ordering of the vitamin, as suggested by FTIR spectra, or the formation of alginate–cellulose microcapsules [66]. Possibly, the alginate–cellulose structures are visible in the TEM images from the FST sample. On the other hand, another explanation could be an ion exchange mechanism (Ca→Fe) and chemical interactions between the carboxylate groups of alginate and iron Fe^3+^ ions, which tend to bind alginate more strongly than most other trivalent and divalent cations [67–68]. Similar effects have been reported for free ionic species of alginate in the presence of Ag nanostructures [64].

Furthermore, additional confirmation of the formation of the biopolymer ionic cross-linking complex can be inferred from the appearance of specific bands at lower frequencies in the Raman spectra of FS0 and FST. These bands suggest crystallization of goethite oxyhydroxide (α-FeOOH)-like structure with bands at 297, 393 cm^−1^ (E_g_ symmetry), and 498 cm^−1^ related to symmetric bending Fe–OH and symmetric stretching Fe–O–Fe/–OH vibrations [69–76]. These bands overlap with magnetite (Fe_3_O_4_)-related bands at 437 cm^−1^ (E_g_ symmetry), 560 cm^−1^ (T_2g_ symmetry), and 692 cm^−1^ (A_1g_ symmetry) features Fe–O asymmetric and symmetric bending and stretching modes [72–73,77–79].

The structural reorganization within the alginate implies a slight disordering of the crystal structure of iron oxides. This disorder may result from more structural and local point defects and unsaturated bonds, making it more prone to forming an ionic cross-linking complex. In this context, the coexistence of oxyhydroxide with Fe^3+^ ions and magnetite with mixed Fe^2+^ and Fe^3+^ valency creates a polysaccharide-stabilized colloid with polysaccharide chains, preventing the aggregation of colloidal species and keeping the nanoparticles within the hydrogel matrix [80–86].

Raman studies provided information consistent with the TEM results, which allows us to claim that the dominant Fe phase in the FS0 sample is α-FeOOH. The presence of Fe_3_O_4_ also seems to be indisputable; however, the direct localization of this phase remains undefined.

#### XPS results and analysis

**Figure 11:**
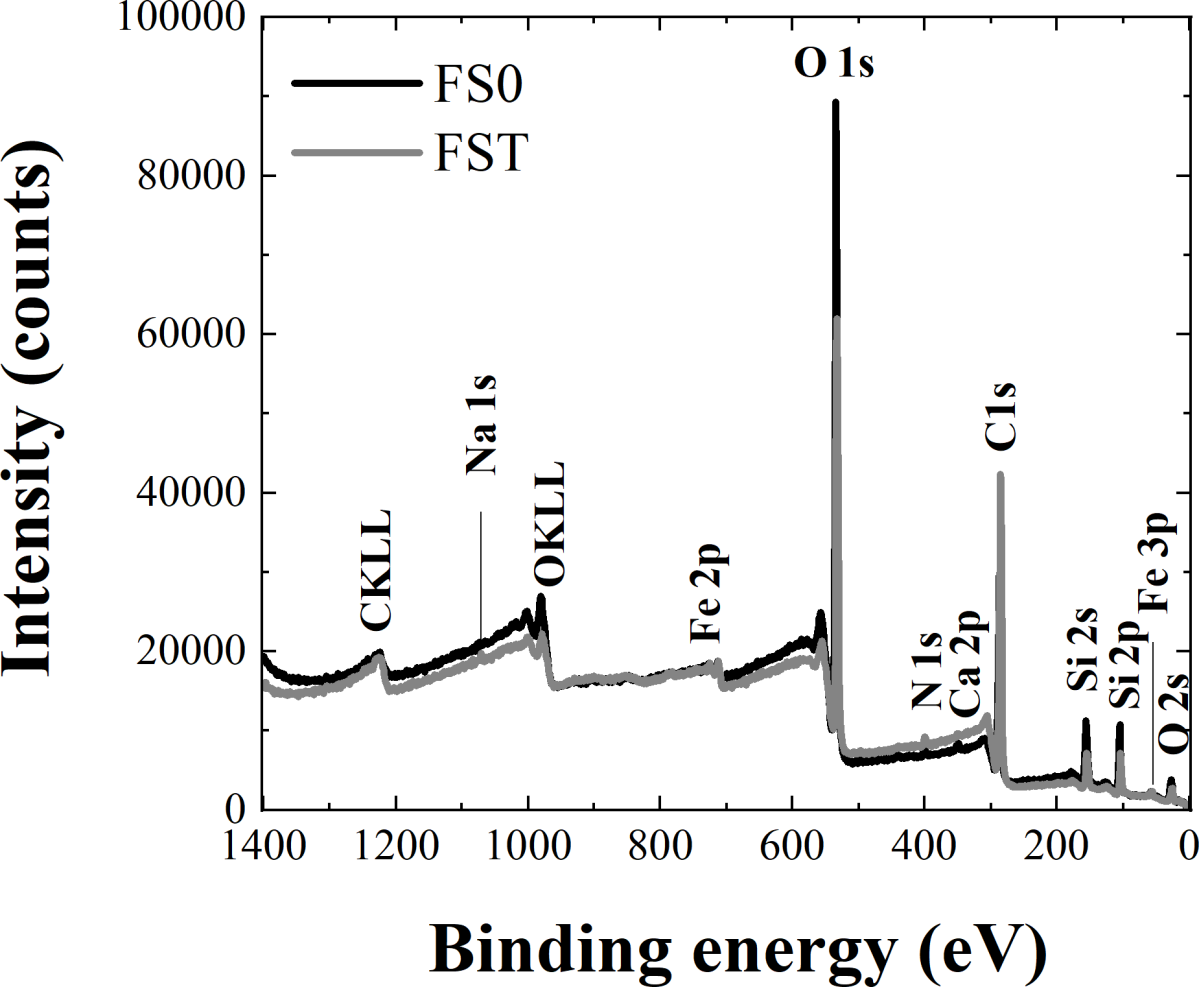
XPS survey spectra for the FS0 and FST samples.

Figure 11 presents the XPS survey spectra for the investigated samples in a wide range of binding energies, where the domination of the O 1s line is indisputable. The discrepancies in the percent atomic composition estimated according to these results are collected in Table 2. In the case of FS0, the majority are O 1s states; next in terms of quantity are C 1s and Si 2p. In the spectrum of the FS0 sample, there was also a small amount <1% of N 1s, Fe 2p, and Ca 2p states, and a trace of Na 1s states. Performed sample dissolution caused the spectrum to be dominated by C 1s, the contribution of Fe 2p and Na 1s states increased, and the Si 2p states contribution decreased almost twofold.

**Table 2:** Atomic composition (%) based on the spectra from Figure 10.

Elements	O	C	N	Fe	Ca	Si	Na

FS0 atomic %	45.97	40.42	0.98	0.84	0.31	11.44	0.06
FST atomic %	31.93	59.08	0.98	1.04	0.07	6.66	0.23

At first glance, according to Table 2, it may be concluded that the dissolution process in physiological saline contributed to the destruction of the calcium alginate shell, as the Ca ion concentration significantly decreased. It should be mentioned that calcium alginate is water insoluble; however, it is slowly dissolved in the solution of sodium chloride, which was registered in the carried out XPS studies. Also, the dissolution process turned out to be not without significance for the concentration of Na ions, whose level increased. It is an effect of salt contamination from physiological saline, which was also proved by the XRD results.

**Figure 12:**
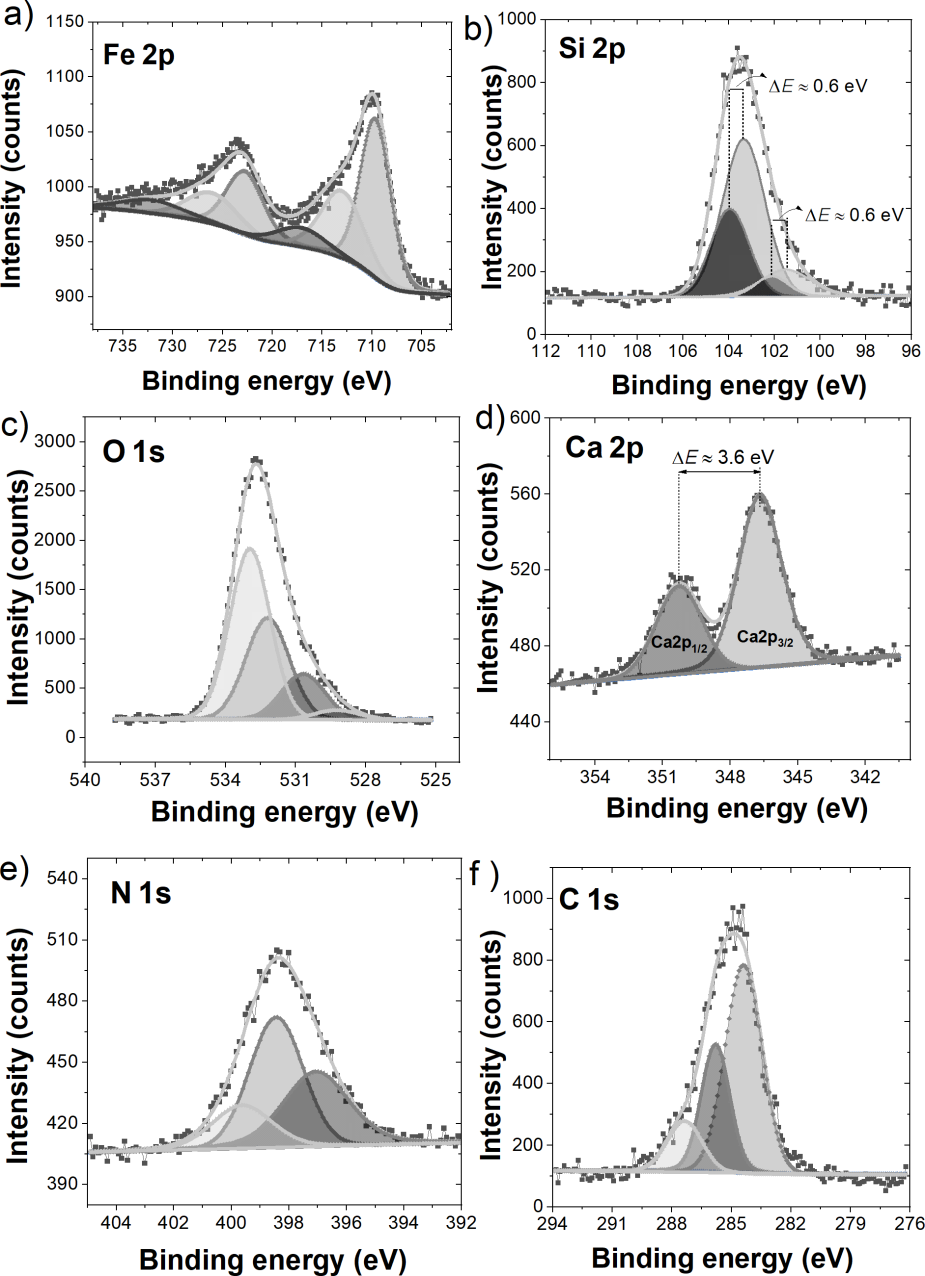
Core level lines registered in the XPS measurements for the FS0 sample. a) Fe 2p, b) Si 2p, c) O 1s, d) Ca 2p, e) N 1s, f) C 1s.

In Figure 11, for all the distinguished states, the high-resolution XPS measurements were performed, the results of which will be further analyzed. Figure 12 presents the spectra for FS0, while the results obtained for FST, due to the similarity with those recorded for FS0, are not presented, but the positions of all registered and deconvoluted peaks for both samples are listed in Table 3. As the most significant for these studies are the iron states, despite their small percentage, the analysis will start with them.

The XPS spectra of the Fe 2p multiplet structure for the FS0 and FST samples exhibit two main peaks with very similar binding energies around 710 eV and 723 eV (see Figure 12a) assigned as Fe 2p_3/2_ and Fe 2p_1/2_ core lines with the spin–orbit splitting of about Δ*E* ≈ 13 eV [87–89]. To the experimental data (grey squares in Figure 12), the fitting procedure was applied, after which the Fe 2p core-level spectra for both samples were deconvoluted into six peaks whose parameters are gathered in Table 3. It is clearly seen that the binding energies of the obtained components under the Fe 2p_3/2_ and Fe 2p_1/2_ envelopes very slightly differ between the samples, which suggests that the dissolution procedure does not change the ionic state of the iron. However, it could only have influence on chemical bonding or coordination of Fe ions [90–91]. For both samples, peaks I and II are characteristic of the Fe^3+^ ion, and their binding energies correspond to literature values for both Fe_2_O_3_ and FeOOH [92]. However, the correlation of these peaks with the Fe_2_O_3_ phase is rejected, as the presence of this phase in the tested samples has not been confirmed by any other studies. Also, in the obtained Fe 2p core level lines there is no trace of Fe^2+^ ions recognized in the Raman studies of Fe_3_O_4_, as its binding energy is substantially lower than those registered here [87,92–93]. It should be stressed that the Fe 2p spectra and the results of their deconvolution obtained in this study are extremely similar to the results for FeOOH from [90], which additionally confirms the presence of one of the iron oxyhydroxide polymorphs in the investigated samples. Analyzing the individual components of the Fe 2p core lines, peaks I most probably are related to the Fe–O–Fe bonds, while peaks II could be a representation of Fe–OH bonds, not necessarily only at the surface [90,93]. The peaks named as III and VI were recognized as the so-called satellite peaks to the peaks I and III, respectively. This is due to the fact that the energy spacing between them and the fundamental peak is placed within the range 6–9 eV, as it is predicted in the literature of iron oxide minerals [87–88,93–94].

**Table 3:** Parameters obtained in the deconvolution process of the XPS spectra of FS0 and FST samples.

Line/peak number	Peak position *E*_B_ (eV)		FWHM (eV)		% Gauss		Area (%)	

Fe 2p	FS0	FST	FS0	FST	FS0	FST	FS0	FST

I	709.67	709.97	3.61	3.59	87	100	35.73	31.46
II	712.99	712.83	5.01	6.01	80	100	21.68	28.90
III	717.13	718.69	5.71	4.82	87	100	6.92	3.83
IV	722.77	723.07	4.38	3.63	80	80	17.86	15.73
V	726.09	725.93	5.98	5.01	100	97	10.84	14.45
VI	732.08	731.13	7.43	6.43	60	80	6.96	5.64

Si 2p								

I	101.49	101.20	2.58	2.46	90	70	11.53	7.66
	102.10	101.81	1.70	2.70	90	70	5.77	3.83
II	103.33	103.26	2.20	2.20	100	100	55.14	59.00
	103.94	103.87	1.97	2.04	100	100	27.57	29.50

O 1s								

	529.25	529.64	2.20	2.20	100	80	3.02	6.77
	530.69	531.48	2.20	2.20	100	100	14.35	29.23
	532.20		2.20		100		31.91	
	532.96	532.96	2.03	2.08	94	97	50.72	64.01

Ca 2p								

	346.67		2.25		80		66.67	
	350.27		2.38		96		33.33	

C 1s								

0		283.64		2.20		80		21.20
I	284.39	284.84	2.20	1.70	100	80	58.06	54.90
II	285.80	286.09	1.70	1.70	90	90	28.85	13.94
III	287.35	287.47	2.05	2.47	100	100	13.09	9.96

Na 1s								

0	397.06		2.70		70		36.22	
I	398.44	398.18	2.20	2.49	96		45.61	42.78
II	399.62	399.65	2.45	2.20	80		18.17	57.22

In addition, the XPS measurements, as a very powerful and sensitive method, confirmed the trace of Si ions in the composition of the samples. Obtained Si 2p spectra are in Figure 12b, while the peak binding energies calculated in the decomposition procedure are collected in Table 3. For both samples, two pairs of peaks can be distinguished: the first, named I, with the binding energy in the range of 101–102 eV, and the second, named II, in the range of 103–104 eV. As the difference in the binding energies in each pair is constant and equal to 0.6 eV, it was concluded that this is a reflection of the spin–orbit splitting of the Si 2p spectrum into 2p_3/2_ and 2p_1/2_ lines [94]. Peaks II are widely described in the literature and are assigned to Si ions in the environment of four oxygen ions, so SiO_2_ [95–97], while peaks I with lower binding energies are most probably related to different numbers of oxygen atoms bound to Si atoms (most likely only two) [96,98]. The correlation of peaks I to SiO should be excluded as the binding energy of the bulk Si line is lower than 100 eV [95]. According to the drug leaflet of the investigated material, silicon dioxide plays the role of an anti-caking agent. Thus, any interaction with other components should be excluded, and only a trace of it was expected in the investigated samples. However, as the Si 2p XSP spectra show, this is not the case, so assuming that the valency of the Si ions is four, some parts of the silicon atoms have an environment other than just oxygen. It appears impossible to determine what other atoms besides oxygen may be bonded to the Si atom to meet the bonding requirements, and only some suggestions may be made. Based on [99], it could be any combination of C and H atoms, whose quantity in the investigated samples is high due to the presence of sucrose and calcium alginate. It should also be added that Si 2p lines are observed for both samples, which is no surprise as silicon dioxide is insoluble in water.

Figure 12c presents the XPS spectral lines of the O 1s states for the FS0 sample, which are asymmetric but with a similar binding energy of around 533.5 eV. From the fit of the O 1s envelops, four bands were obtained for the FS0 sample, while for FST, only three bands were obtained. All calculated parameters are presented in Table 3. However, identifying the origin of these peaks and assigning them to specific compounds seems impossible because virtually every component of the sample contains oxygen bonds, and their binding energies can be indistinguishable. In this context, we can only propose some predictions. The peaks with the lowest binding energy, 529.25 eV for the FS0 sample and 529.64 eV for FST, are rather assigned to the lattice oxide (i.e., Fe–O–Fe bonds) while the next peaks on the energetic scale could be related to the hydroxyl species, among others of the iron hydroxide, carboxylic group, Si–O bonds, or even absorbed water molecules by calcium alginate and cellulose, respectively [88,96–98,100]. Conversely, the increase of the 532.96 eV peak area after dissolution was registered, and it seems quite likely that it is the reflection of water adsorption by calcium alginate and cellulose.

Based on the recorded O 1s spectra, it was impossible to determine which of the iron oxyhydroxide polymorphs is present in the investigated material, if at all, given that the binding energies for O 1s states predicted in the literature are very similar for all Fe(III) oxyhydroxides. In addition, in these samples, we can distinguish other substances containing this type of bond. However, the observation of the OH^−^ line in the O 1s spectra is a strong proof of the presence of iron oxyhydroxide in the investigated materials.

The XPS spectrum for the Ca 2p line measured from the FS0 sample is plotted in Figure 12d, where two well-separated peaks are visible, specified as Ca 2p_3/2_ around 346.67 eV and Ca 2p_1/2_ at 350.27 eV. It can be deduced from the sample composition that the registered Ca 2p line has a source in the Ca ions contained in calcium alginate. This is also confirmed by the mention earlier in the text about the solubility of calcium alginate in the solution of water and NaCl.

The N 1s and C 1s lines presented in Figure 12e,f also deserve a comment. Among the substances in the sample composition, nitrogen atoms are contained in vitamins B2, B6, B12, and B9; thus, the N 1s spectrum is most probably their reflection. However, bearing in mind the fact that carbon forms numerous bonds with nitrogen in the vitamin structure, those two spectra will be analyzed together. The N 1s spectra appear to be symmetrical, in contrast to the C 1s lines, which broaden into the lower binding energies. From the deconvolution procedure, we obtained three components of both N 1s and C 1s lines for the FS0 sample and two and four components of these lines, respectively, for the FST sample. The binding energies of these peaks are collected in Table 3.

Analyzing the N 1s spectrum for both samples, we relate the peaks at 398.44/398.18 eV for FS0/FST, called I, to C–N bonds, while the peaks at 399.62/399.65 eV for FS0/FST, called II, to C=N bonds, as these two types of chemical bonds are expected for the vitamins specified above, especially the latter are well known and assigned to the pyridine ring [101–103]. However, for sample FST, the assignment of the N 1s peak at a binding energy of 397.06 eV, called 0, is not so obvious, and the only suggestion which could be made is that it may be a trace of vitamin B9 [104]. This idea seems even more likely since we do not see this line in the results of the dissolved sample.

The interpretation of the C 1s envelopes, in analogy to the O 1s spectra, turns out to be complex since carbon occurs in all sample components except in the iron compound and silicone dioxide. Considering the C 1s spectra for both samples, we may attribute the peaks I to C–C bonds present in vitamin B structures [100–101,103], cellulose [105–106], calcium alginate [107], and sucrose [108]. However, the source of peaks I may be C=C [103] and/or C–H [101] bonds distinguished in N-containing organic polymers, meaning here B vitamins. The binding energy of peaks II agrees quite well with that of C=N bonds for the pyridine rings [101–103], whereas peaks III agrees with that of C–N bonds from B vitamin structures [102–103]. In addition, for the sample FST, the peak at 283.64 eV, named as 0, is well visible, which may be assigned to carbon beta (Cβ) in the cellulose structure [105].

Since all the above vitamins are so-called water soluble, the lack of differences in the quantity of N atoms in the atomic composition of the samples and the increase in the quantity of C atoms (Table 2) after dissolution turned out to be an unexpected result. Here, the interpretation of this phenomenon is proposed. According to C–C bonds, as they are distinguished, in the water-insoluble cellulose the presence of peak I in C 1s spectrum is understandable. This also applies to the intensity of these peaks since the cellulose is a filling substance in the investigated material; thus, its quantity is significantly higher compared to the amount of microcapsules and B vitamins. Peaks II in the C 1s and N 1s spectra, most probably related to the C=N bonds in the pyridine rings, were preserved after dissolution as pyridine is a water-miscible substance. Conversely, the appearance of the Cβ peak in the dissolved sample indicates that the dissolution process had an impact on the composition of the sample and the structure of its ingredients, which is also confirmed above by the other measurements performed here. However, precise identification of the changes that have occurred in the structure and composition seems impossible to explain according to the XPS results.

#### EPR results and analysis

**Figure 13:**
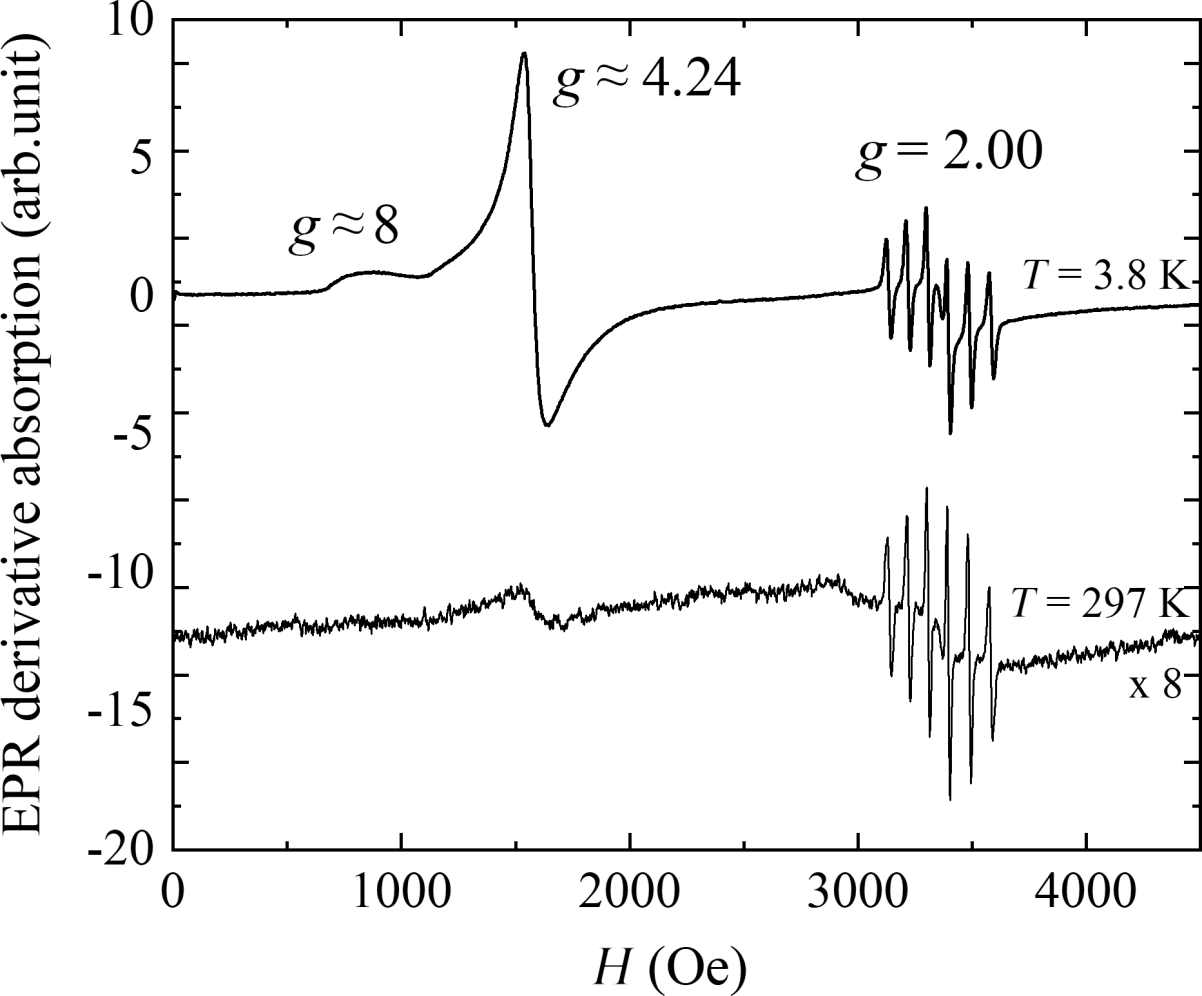
EPR spectra recorded at 3.8 and 297 K for the FST sample.

Figure 13 presents the EPR derivative absorption spectra performed at 297 K and 3.8 K for the FST sample. The observed spectrum is a superposition of the resonance patterns from different sources. The first and most intense pattern comes from wide continuous absorption visible in the field range of about 800–3500 Oe. This absorption has three distinctive spectral density centered around *H* ≈ 850, 1580, and *H* = 3350 Oe (that corresponds to the effective g-factors of about 8, 4.24, and 2.00, respectively). It is well-known in the literature that resonance absorption is attributed to the averaged fine structure of Fe^3+^ ions [109–111]. The absorption around *H* ≈ 3350 Oe, besides having a wide signal from Fe^3+^, has two additional resonance patterns. Those are six thin equidistant lines centered around *g* = 2 attributed to the Mn^2+^ ions [110,112]. Finally, the third pattern represented by a single thin line at *g* = 2.001 is usually attributed to unpaired electrons associated with electronic defects/organic free radicals [109]. The number of free radicals estimated from the integral intensity of the thin line is ≈10^14^ spins (per sample). Upon lowering the temperature, the intensity of the resonance lines with the effective *g*-factor = 4.24 and ≈8 roughly increases following the Curie law, meaning that absorption comes from magnetically isolated ions.

The variety of the resonance lines attributed to Fe^3+^ indicates inhomogeneously bound iron in the FST sample. Close to cubic surrounding, the S-ion of Fe^3+^ (3d^5^ electronic configuration) gave rise to a wide resonance line with close to free ion *g* value (2.0023). The resonance line with effective *g*-factor ≈4.24 and ≈8 are assigned to paramagnetic Fe^3+^ ions in the distorted, lower than cubic symmetry of the ligand field. The former line is well-known for rhombically distorted positions of high-spin state Fe^3+^ ion, and it arises from partially allowed transition within the heavily mixed 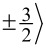 Kramers doublet otherwise forbidden by the selection rule (Δ*m*_s_ = 1), while the latter is attributed to the transition between 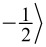 and 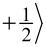 spin states for axially distorted Fe^3+^ sites [109,113]. However, the environment of the Fe^3+^ ions which are the sources of these resonance is not so obvious, as several interpretations can be found in the literature. Some authors link the line at the effective *g*-factor ≈4.2 to an octahedral environment of high-spin state Fe^3+^ ions [114–115], whereas other to a tetrahedral one [109,116]. In addition, there are also works where the authors propose both of them with the rhombic distortion [117–118]. For the FST sample, the last case fits best because both environments are possible since the undissolved FS0 sample contains, besides FeOOH, also Fe_3_O_4_, and it can be suspected that the dissolution procedure modified the surroundings of the Fe^3+^ ions. The situation of the resonance with effective *g*-factor ≈8 is more clear, which most probably reveals the Fe^3+^ ions in axial symmetry sides [109,113,119].

It should be added that the broad line with *g* = 2 obscured by the sextet seems to have a source in the exchange-coupled Fe^3+^ ions into pairs or clusters [114,116]. This explanation seems more likely as the presence of the interacting iron ions in the FST sample was confirmed by dc and ac magnetic studies. A very interesting result of the EPR study is the observation of the sextet at *H* = 3350 Oe, correlating with the nonintentional dopant of Mn^2+^ ions. It should be excluded that Mn impurities are the result of the dissolution process, which means that they are present also in the undissolved sample. It should be stressed that manganese was not registered by any other method used in this study proving the extraordinary sensitivity of EPR and it usefulness in drug examinations.

Summarizing, the results of EPR studies agree well with the results of dc and ac magnetic measurements performed on the FST sample. The obtained results are further evidence that the dissolution process has ended with the decomposition of the microcapsules and nanoparticles.

## Conclusion

The present study shows a multi-technique characterization of microencapsulated iron sucrose and the evolution of its structural and physical properties with the dissolution time. The structural studies revealed that the investigated compound consists of calcium alginate microcapsules, filled with FeOOH (core)/sucrose (shell) nanoparticles, and embedded in a mixture of micro-cellulose, vitamins and SiO_2_. Results from TEM, Raman, and XPS suggest that the most possible form of iron oxyhydroxide in the nanoparticle core is the goethite (α-FeOOH). Magnetic studies were performed such that the nanoparticles are a noninteracting system of grains, which exhibits the blocking process at low temperatures, <*T*_B_> ≈ 10 K, and becomes superparamagnetic above 70 K. This behavior was correlated with the presence of the sucrose shell; whose thickness was sufficient enough to separate the magnetic cores. The net particle magnetic moment obtained in the fitting of the modified Langevin function to the *M*(*H*) relations was estimated as ≈50–70 μ_B_ and originates from uncompensated spins within nanoparticles. The decomposition process of the microcapsule and nanoparticles along with the dissolution time was recorded in measurements of the temperature dependence with magnetization. It is shown as a shift of the maximum associated with the nanoparticle blocking process to lower temperatures and its disappearance for a completely dissolved sample. Differences in FTIR, Raman, and XPS spectra registered for undissolved and completely dissolved samples clearly indicate the structural reorganization of the tested compound after dissolution. The most possible effects were: molecular reorganization of the vitamin structure (also confirmed by the XRD results), formation of alginate–cellulose microcapsules, and formation of Ca, Na, and Fe complexes. The XPS spectra indicate that the ionic state of iron (3+), remained preserved after the dissolution procedure and, unlike the Raman spectra, do not indicate Fe_3_O_4_ impurities. The EPR studies of the completely dissolved sample revealed noninteracting and interacting Fe^3+^ ions, meaning that the dissolution procedure did not lead to the total decomposition of the nanoparticle magnetic cores. Additionally, the EPR measurement was the only one performed to show the presence of manganese, which is a nonintentional dopant. Complete decomposition in contact with physiological salt of the investigate compound even after 12 hours proves that the sucrose shell and additional encapsulation in calcium alginate microcapsules fulfill their role in stabilizing the iron carrier and not allowing its release too quickly. The work presented here shows that magnetic and spectroscopic studies together with structural analysis provide valuable information about iron deficiency drugs. In consequence, these methods can be used in industry for product characterization before its implementation in the market.

## Experimental

### Samples

Presented studies concentrates on the iron sucrose (III) compound contained in the diet supplement called Ferisan^®^ (Solinea Company, Poland). According to the product information, the iron sucrose (III) in Ferisan^®^ has a form called AB-Fortis^®^ iron – special microcapsules developed by AB-Biotics^®^. From the AB-Fortis^®^ technical sheet, it is known that a single microcapsule has a diameter below 20 µm, and consists of iron sucrose as the core, which is covered with a calcium alginate layer [20]. Besides microcapsules rich in iron, the Ferisan^®^ pill includes vitamins (C, B2, B6, B12), folic acid, SiO_2_ as an anticaking agent, and cellulose, hereinafter referred to as additives. In addition, each pill has a coating composed of, among others, dyes based on iron hydroxides. However, the samples used for the presented studies were cut from the internal part of the Ferisan^®^ pill. Thus, the influence of this coating was excluded.

The default sample, called FS0, was not subjected to any processes before the measurements, wheareas the other samples, FS1, FS3, FS7, FS9, FS12, FST, were dissolved in saline (0.9% NaCl water solution). The number in the names of the dissolved samples represents the time (in hours) for the dissolution process. The step-by-step process of dissolving the material was as follows. The uncoated part of the pill was left in saline for the time corresponding to the sample name. The obtained solution was then poured onto laboratory paper, and left to dry for 48 h at room temperature. For the sample FST, the time of dissolution was infinite, which means that the uncoated part of the pill was placed into physiological salt and allowed to self-evaporate and self-dry for a week.

### Methods

#### Structural properties

Morphology, structure, and composition were tested using powder X-ray diffraction, scanning and transmission electron microscopy. X-ray diffraction patterns were registered for samples FS0 and FST using a X’Pert Pro Alpha1 MPD diffractometer (Panalytical) (λ = 1.5406 Å). Morphology of the FS0 sample was examined on a high-resolution SEM-Auriga microscope (Carl Zeiss). Other samples were not tested via SEM due to the charging effect, which made observation significantly difficult. The TEM observations were carried out for the samples FS0 and FST by a high-resolution JEOL JEM3010 transmission electron microscope. For TEM measurements, the sample solutions were dispersed in distilled water and dropped onto a carbon support grid. The chemical compositions of the nanocrystallites were determined by the Elite-T TEM EDAX/GATAN SDD 70 mm^2^ EDS System attached to the JEOL JEM3010 TEM.

#### Magnetic properties

All the samples distinguished in the introduction were examined using the Physical Property Measurement System (PPMS, Quantum Design) with the vibrating sample magnetometer option (VSM) – enabling DC magnetic measurements, and ACMS option for measuring dynamic magnetic properties. Studies performed with VSM included the measurements of temperature curves, *M*(*T*), and magnetization curves, *M*(*H*). *M*(*T*) relations, were collected in the well-known ZFC–FC regimes [120], in the temperature range from 2 to 300 K at a stable external magnetic field selected in the range from 50 Oe to 50 kOe. Conversely, *M*(*H*) curves were measured at ±90 kOe in sweep mode with a speed of 100 Oe/s at several stable temperatures selected according to the *M*(*T*) dependences. The results of the AC measurements were temperature dependences of the real and imaginary parts of the magnetic susceptibility, χ’(*T*) and χ’’(*T*) respectively. These dependences were recorded for an alternating magnetic field amplitude equals to 10 Oe at several frequencies, *f*, ranging from 10 Hz to 10 kHz. In the text, the χ’(*T*) and χ’’(*T*) results are shown only for selected frequencies.

#### Spectroscopic properties

Fourier-transform infrared spectroscopy and Raman scattering were applied to phase composition, as well as the impact of iron on the structural modification of the FS0 and FST nanocomposites.

The FTIR spectra were measured on an Agilent Cary 640 spectrometer (Agilent Technologies, Inc. Headquarters, Santa Clara, CA, United States) equipped with a standard source and DTGS Peltier-cooled detector, using the ATR method and a Ge plate accessory in the 900–4000 cm^−1^ range. Spectra were recorded by accumulating 32 scans with a spectral resolution of 4 cm^−1^ and then subjected to post-processing analysis to the baseline, water, and carbon dioxide correction. Finally, ATR corrections were done using the refractive index of the iron oxide (*n* ≈ 2.3435). Raman spectra were obtained with a WITec confocal Raman microscope CRM alpha 300 R (WITec Wissenschaftliche Instrumente und Technologie GmbH, Ulm, Germany) equipped with an air-cooled solid-state laser (λ = 532 nm, *P* = 2 mW). The excitation laser radiation was coupled into a microscope through a polarization-maintaining single-mode optical fiber with a 50 μm diameter, and the monochromatic light was focused on the sample by an air Olympus MPLAN (100×/0.90NA) objective. Raman scattered light was passed through a multi-mode fiber (50 μm diameter) into a monochromator with a 600 line/mm grating and a CCD camera. The spectrometer monochromator was checked before the measurements using a silicon plate (520.7 cm^−1^). Raman spectra (RS) in the 150–4000 cm^−1^ region were accumulated by 60 scans with an integration time of 10 s and a resolution of 3 cm^−1^, while the post-processing analysis, such as baseline correction or cosmic ray removal, was done in the WITecProjectFive Plus (version 5.1.1, WITec Wissenschaftliche Instrumente und Technologie GmbH, Ulm, Germany). Finally, the peak fitting analysis of FTIR and RS data was performed using the GRAMS (version 9.2, Thermo Fisher Scientific, Waltham, MA, USA) software.

X-ray photoemission spectroscopy data were measured for FS0 and FST at room temperature by using a monochromatic X-ray source Al Kα (1486.6 eV). The samples were fixed into the sample holder by double-coated conductive carbon tape and subsequently stored under an ultra-high vacuum in the storage chamber for at least 24 h prior to being measured in the main chamber. All the XPS spectra obtained were calibrated using a C 1s peak having a binding energy of BE = 284.8 eV as a carbon peak, usually originating from the carbon adsorbed at the surface of the sample and used as a reference for charge correction. The deconvolution of core level lines was performed in the MultiPak 9.8 software by the combination of the Gauss–Lorentz function and Shirley background using the doublet option for all analyzed 2p lines.

For the FST sample, electron paramagnetic resonance was used, and the EPR spectra were collected at 3.8, 190, and 297 K using an X-band (9.382 GHz) EMX Bruker ER083CS spectrometer.

## Data Availability

Data generated and analyzed during this study is available from the corresponding author upon reasonable request.
